# Engineered *Rhodobacter capsulatus* as a Phototrophic Platform Organism for the Synthesis of Plant Sesquiterpenoids

**DOI:** 10.3389/fmicb.2019.01998

**Published:** 2019-09-06

**Authors:** Katrin Troost, Anita Loeschcke, Fabienne Hilgers, Armagan Yakup Özgür, Tim Moritz Weber, Beatrix Santiago-Schübel, Vera Svensson, Jennifer Hage-Hülsmann, Samer S. Habash, Florian M. W. Grundler, A. Sylvia S. Schleker, Karl-Erich Jaeger, Thomas Drepper

**Affiliations:** ^1^Institute of Molecular Enzyme Technology, Heinrich Heine University Düsseldorf, Düsseldorf, Germany, Forschungszentrum Jülich GmbH, Jülich, Germany; ^2^Central Institute for Engineering, Electronics and Analytics ZEA-3, Analytics, Forschungszentrum Jülich GmbH, Jülich, Germany; ^3^INRES-Molecular Phytomedicine, Rhenish Friedrich–Wilhelm University of Bonn, Bonn, Germany; ^4^Institute of Bio- and Geosciences IBG-1, Biotechnology, Forschungszentrum Jülich GmbH, Jülich, Germany

**Keywords:** terpenoid, natural product, valencene, patchoulol, *Rhodobacter capsulatus*, metabolic engineering

## Abstract

Sesquiterpenoids are a large class of natural compounds offering manifold properties valuable for food, cosmetics, agriculture, and pharma industry. Production in microorganisms is a sustainable approach to provide sesquiterpenoids for research and industrial use independent of their natural sources. This requires the functional transfer of the respective biocatalytic pathways in an adequate host microorganism offering a sufficient supply of precursors that is ideally adjusted to the individual demand of the recombinant biosynthesis route. The phototrophic purple bacterium *Rhodobacter capsulatus* offers unique physiological properties that are favorable for biosynthesis of hydrophobic terpenes. Under phototrophic conditions, it develops a large intracytoplasmic membrane suitable for hosting membrane-bound enzymes and metabolites of respective biosynthetic pathways. In addition, *Rhodobacter* harbors an intrinsic carotenoid biosynthesis that can be engineered toward the production of foreign terpenes. Here, we evaluate *R. capsulatus* as host for the production of plant sesquiterpenoids under phototrophic conditions using patchoulol and valencene as a proof of concept. The heterologous expression of patchoulol synthase PcPS from *Pogostemon cablin* as well as the valencene synthases CsVS from *Citrus sinensis* and CnVS from *Callitropsis nootkatensis* led to the production of the respective sesquiterpenoids in *R. capsulatus*. To analyze, if gradually adjustable formation of the key precursor farnesylpyrophosphate (FPP) is beneficial for sesquiterpene synthesis under phototrophic conditions, the intrinsic 1-deoxy-D-xylulose 5-phosphate (DXP) pathway genes as well as the heterologous mevalonate pathway genes were modularly expressed in various combinations. To this end, different plasmids and chromosomally integrated expression tools were developed harboring the strong and tightly controlled P*_*nif*_* promoter for heterologous gene expression. Notably, comparative studies identified a distinct combination of precursor biosynthetic genes as best-performing setup for each of the tested sesquiterpene synthases. In summary, we could demonstrate that *R. capsulatus* is a promising alternative platform organism that is suited for sustainable sesquiterpenoid formation under phototrophic cultivation conditions. A modular engineering of *R. capsulatus* strains via tailored co-expression of FPP biosynthetic genes further allowed adaptation of sesquiterpene precursor formation to its catalytic conversion by different plant terpene synthases.

## Introduction

The class of terpenoid secondary metabolites comprises over 80,000 structurally diverse compounds, a majority of which was isolated from plants (Bian et al., 2017; Christianson, 2017; Pemberton et al., 2017). In fact, terpenoids represent one of the main groups of secondary metabolites with diverse biological functions and valuable properties for various industrial applications (Kallscheuer et al., 2018). Terpenoids are basically divided into different terpene classes based on the number of carbon atoms: hemi- (C5), mono- (C10), sesqui- (C15), di- (C20), tri- (C30), tetra- (C40), and polyterpenes (>C40) (Ruzicka, 1953; Croteau et al., 2000). All terpenoids are biosynthesized from the isoprene C5 scaffolds isopentenyl pyrophosphate (IPP) and dimethylallyl pyrophosphate (DMAPP), which are built via the mevalonate (MVA) pathway or the 1-deoxy-D-xylulose 5-phosphate (DXP) pathway, also called 2-*C*-methyl-D-erythritol 4-phosphate (MEP) pathway. The MVA pathway, which uses acetyl-CoA as substrate, is mainly present in eukaryotes (mammals, plants and fungi), but also archaea and a few bacteria (Boucher and Doolittle, 2000); the DXP pathway, which starts from glyceraldehylde-3-phosphate (GAP) and pyruvate, is particularly used by bacteria, cyanobacteria, and green algae (Frank and Groll, 2017). The latter also occurs in plants where it is located in the plastids while the MVA pathway is cytosolic (Dewick, 2002). IPP and DMAPP represent the starting molecules for the biosynthesis of elongated linear prenyl pyrophosphates in subsequent head-to-tail condensations catalyzed by prenyltransferases, yielding C10 geranyl pyrophosphate (GPP), C15 farnesyl pyrophosphate (FPP), and C20 geranylgeranyl pyrophosphate (GGPP). GPP serves as precursor for monoterpenoids, FPP for sesqui- and triterpenoids and GGPP for di- and tetraterpenoids.

The structurally highly diverse terpenoids naturally fulfill manifold functions, including photoprotection (carotenoids), repellant activity against predators and parasites (e.g., verbenone), communication (e.g., pinene), regulation of the membrane fluidity (bacterial hopanoids, eukaryotic sterols), electron transfer in the respiratory chain and photosynthesis (ubiquinone and plastoquinone), or hormone activity (e.g., gibberellins) (Langenheim, 1994; Gershenzon and Dudareva, 2007; Pichersky and Raguso, 2016). Due to their bioactive properties, some terpenoids can be utilized as agents that are effective against pathogens, inflammations, or cancer (Efferth, 2017; Schempp et al., 2018). Moreover, many are applicable as flavors and fragrances in the food and cosmetics industry such as the sesquiterpenoids patchoulol and valencene. Due to its characteristic earthy and woody scent, patchoulol is one of the most prominent fragrances for the perfume and cosmetic industry (Bauer et al., 2001). Valencene is commercially used to add its citrus flavor to beverages (Schempp et al., 2018). In the past, these compounds were exclusively obtained from natural plant sources. Patchoulol was extracted from the Indian patchouli *Pogostemon cablin* and valencene from different citrus species like *Citrus sinensis*. However, the biotechnological production in microbial hosts can represent an ecologically favorable, cost-effective and sustainable alternative production route (Marienhagen and Bott, 2013; Kallscheuer et al., 2018; Schempp et al., 2018). In fact, large-scale biotechnological production has been demonstrated for some prominent terpenoids, for example artemisinic acid, which is the precursor of the antimalaria agent artemisinin (Paddon et al., 2013). Furthermore, different industrial terpenoid flavor and fragrance compounds produced by engineered microorganisms, including β-farnesene, valencene, nootkatone and patchoulol, are nowadays marketed by the companies Amyris, Evolva, Isobionics, and Firmenich, respectively (Schempp et al., 2018).

Since appropriately high product titers are essential for the development of a bioeconomically feasible production process, one research focus is the engineering of microbial host metabolism and target pathways (Kirby and Keasling, 2008; Mitchell, 2011; Chen et al., 2015; Bian et al., 2017). The most commonly used microorganisms for the heterologous terpenoid production are *Escherichia coli* and *Saccharomyces cerevisiae*, but also others including phototrophic bacteria are gaining interest, as documented by the *Rhodobacter sphaeroides*-based production of valencene and nootkatone, marketed by Isobionics. The phototrophic non-sulfur purple α-proteobacteria of the genus *Rhodobacter* feature some physiological characteristics which are especially advantageous for terpenoid production: (i) the cell membrane is commonly considered to be a critical determinant in terpenoid production, since it can function as storage compartment for pathway enzymes and hydrophobic compounds (Das et al., 2007; Arendt et al., 2017). In contrast to non-phototrophic microbes, species of the genus *Rhodobacter* can form a highly enlarged intracytoplasmic membrane system (ICM) where the components of the photosynthetic apparatus are naturally housed (Tucker et al., 2010; Drews, 2013). The ICM thus constitutes a naturally enlarged reservoir for membrane-embedded enzymes and metabolites. (ii) Relying on the DXP pathway for precursor supply, these phototrophic bacteria synthesize the carotenoids (i.e., tetraterpenoids) spheroidene and spheroidenone (Armstrong et al., 1989; Armstrong, 1997), thereby offering a robust and effective isoprenoid metabolism as basis for engineering the host’s metabolism toward recombinant terpenoid production. (iii) *Rhodobacter* species are capable of growing photo(hetero)trophically in inexpensive minimal media at relatively high growth rates, allowing to use sunlight as energy source within sustainable cultivation processes.

Previous studies showing the production of triterpenoids in *R. capsulatus* (Khan et al., 2015), and the sesquiterpenoid valencene in *R. sphaeroides* (Beekwilder et al., 2014) have indicated that heterologous terpenoid production can be optimized by engineering the isoprenoid precursor biosynthesis. Here, co-expression of an FPP synthase (IspA) and rate-limiting enzymes of the DXP pathway DxS synthase and IPP isomerase (Idi) (Khan et al., 2015) as well as the introduction of the MVA pathway (Beekwilder et al., 2014), which does not naturally occur in *Rhodobacter*, were initially demonstrated to enhance recombinant terpenoid production.

In this study, we therefore aimed to investigate if modular co-expression of DXP/MVA genes by the strictly controlled P*_*nif*_* promoter can help to reconstitute plant sesquiterpenoid pathways in *R. capsulatus.* To analyze terpenoid formation under phototrophic growth conditions, patchoulol and valencene synthases from different plants were used as an example.

## Materials and Methods

### Bacterial Strains and Cultivation Conditions

*Escherichia coli* strain DH5α (Hanahan, 1983) was used for cloning and strain S17-1 (Simon et al., 1983) for conjugation. *E. coli* was cultivated at 37°C on LB agar plates or in liquid LB medium (Luria/Miller, Carl Roth^®^), supplemented with 50 μg/mL kanamycin, 10 μg/mL gentamicin or 10 μg/mL tetracycline when appropriate. *R. capsulatus* SB1003 (Strnad et al., 2010) was used for heterologous terpene production. The wildtype strain and derivatives thereof were cultivated on PY agar plates (Klipp et al., 1988) containing 2% (*w*/*v*) Select Agar (Thermo Fisher Scientific) or in RCV liquid medium (Weaver et al., 1975) at 30°C, both supplemented with 25 μg/mL rifampicin. For strain SB1003-MVA, additional 4 μg/mL gentamicin were used. Cultivation was conducted under anaerobic photoheterotrophic conditions and permanent illumination with bulb light (2500 lx).

### Construction of Strain SB1003-MVA

For construction of strain *R. capsulatus* SB1003-MVA, a vector carrying the MVA gene cluster as interposon cassette was constructed. To this end, chromosomal sequences upstream (*Nde*I-*crp*’-*fdxD-nifH*’-*Xba*I, 1.5 kb) and downstream (*Kpn*I-“*nifK-nifU1-rpoN*”-*Xho*I-*Eco*RI, 1.5 kb) of the *nifHDK* operon of *R. capsulatus* SB1003 were PCR-amplified and cloned into vector pUC18 to create vector pUC18-nifupdown. The MVA pathway encoding gene cluster from *Paracoccus zeaxanthinifaciens* ATCC 21588 (*Xba*I-*mvaA-idi-hsc-mvk-pmk-mvd-Nhe*I/*Kpn*I, 6.4 kb) was amplified by PCR using genomic DNA as template. A gentamicin resistance gene with the respective promoter (*Nhe*I-*aacC1-Kpn*I, 0.8 kb) and a mob-Tc cassette (*Xho*I-*oriT-tetR-Xho*I, 2.6 kb) were PCR-amplified using vector pIC20H-RL (Loeschcke et al., 2013) as template. The MVA pathway genes and *aacC1* were cloned successively into pUC18-nifupdown between the chromosomal up- and downstream sequences of the *nifHDK* operon, and the mob-Tet cassette was added aside that to construct vector pMVA-int-Pnif. This construct was transferred to *R. capsulatus* by conjugation. Among exconjugants, clones carrying the MVA gene cluster in the chromosome were identified by use of gentamicin-supplemented medium. Replica-plating on tetracycline and gentamicin-supplemented medium showed that the strain was exclusively obtained with single-cross-over integration. Therefore, the strain was cultivated on gentamicin-containing medium to ensure stability of the integration cassette.

### Construction of Expression Vectors

The expression vector pRhon5Hi-2 was cloned using pRhotHi-2 (Katzke et al., 2010) as respective backbone. For the construction, genomic DNA of *R. capsulatus* was isolated as a template for PCR. For the amplification of the 401-bp P*_*nif*_* DNA fragment (NCBI Genbank Accession MG208548 deposited by Özgür and coworkers), primers Pnif-fw and Pnif-rv were used harboring an *Nhe*I and *Xba*I site, respectively. After hydrolyzation, the P*_*nif*_* fragment was cloned into the *Nhe*I and *Xba*I sites of vector pRhotHi-2, thereby substituting the original T7 promoter. The sequences of patchoulol synthase PcPS from *P. cablin* (UniProt: Q49SP3.1), the valencene synthases CnVS from *Callitropsis nootkatensis* (GenBank: AFN21429.1) and CsVS from *Citrus sinensis* (Uniprot: Q71MJ3) were used to generate DNA sequences with suitable codon-usage for expression in *R. capsulatus* with the help of the Codon Optimization Tool by IDT Integrated DNA Technologies and the Graphical Codon Usage Analyzer tool (Fuhrmann et al., 2004). Genes were obtained as synthetic DNA by Eurofins Genomics (*PcPS*, 1.7 kb; *CsVS*, 1.6 kb; and *CnVS*, 1.8 kb) flanked by appropriate restriction endonuclease recognition sequences (*Nde*I/*Hin*dIII). The genes encoding IspA from *R. capsulatus* SB1003 (*ispA*, 0.9 kb), 1-deoxy-D-xylulose-5-phosphate synthase and IPP isomerase from *Rhodobacter sphaeroides* 2.4.1 (*dxs*, 1.9 kb; *idi*, 0.5 kb) and the gene cluster encoding the MVA biosynthesis pathway from *Paracoccus zeaxanthinifaciens* ATCC 21588 (*mvaA-idi-hsc-mvk-pmk-mvd*, 6.3 kb) were amplified using the respective genomic DNA as template. Suitable restriction endonuclease recognition sequences were added via the oligonucleotide primers for the following procedures: all genes were cloned into expression vector pRhon5Hi-2, enabling an induction of target gene expression via the provided nitrogen source. Terpenoid synthase (TPS) encoding genes, namely *PcPS*, *CsVS*, *CnVS*, were cloned into the vector as *Nde*I/*Hin*dIII fragments, creating pRhon5Hi-2-PcPS/CsVS/CnVS, respectively. The TPS genes are thereby placed immediately downstream of the P*_*nif*_* promoter and RBS of the vector. To generate vectors carrying one additional isoprenoid biosynthetic gene or the MVA gene cluster, i.e., with the architecture pRhon5Hi-2-TPS-ispA/dxs/idi/MVA, the different PCR products were cloned into the three variants of pRhon5Hi-2-TPS as *Hin*dIII/*Xho*I fragments. For the construction of the expression cassettes carrying combinations of increasing length downstream of the TPS genes with the structure *TPS-ispA-dxs*/*TPS-ispA-dxs-idi*/*TPS-ispA-dxs-idi-MVA*, the PCR products of *ispA*, *dxs*, *idi* and the *MVA* cluster were cloned successively into the vector pRhon5Hi-TPS as *Hin*dIII/*Xho*I, *Mlu*I/*Xho*I, *Spe*I/*Xho*I, *Kpn*I/*Xho*I fragments, respectively. A scheme of the described strategies for cloning of expression vectors, nucleotide sequences of expressed sesquiterpenoid synthases and used primers are summarized in Supplementary Figure S1 and Supplementary Tables S1, S2.

### Heterologous Production of Plant Sesquiterpenoids Valencene and Patchoulol in *R. capsulatus*

For the expression of the heterologous genes in *R. capsulatus*, respective pRhon5Hi-2-based plasmids were transferred to the host via conjunctional transfer employing *E. coli* S17-1 as donor as previously described (Klipp et al., 1988). Thereafter, exconjugants were selected and further cultivated on PY agar, containing 25 μg/mL kanamycin and 25 μg/mL rifampicin. Subsequently, photoheterotrophic cultivation was conducted in liquid RCV medium containing 25 μg/mL kanamycin and 25 μg/mL rifampicin in airtight Hungate tubes (Hungate, 1969): pre-cultures of 15 mL RCV medium containing 0.1% (NH_4_)_2_SO_4_ were inoculated with cells from agar plates and incubated for 48 h. Expression cultures were inoculated from pre-cultures to an initial OD_660__nm_ of 0.05 in 14 mL RCV medium containing 0.1% serine as exclusive nitrogen source. The absence of ammonium together with photoheterotrophic conditions (the absence of oxygen) led to the induction of the P*_*nif*_*-dependent target gene expression. The cultures were overlaid with 500 μL *n*-dodecane for extraction of the heterologously produced terpenoids, and incubated for 5 days without agitation.

### GC-Analysis and Quantification of Sesquiterpenoids

After cultivation of expression cultures, Hungate tubes were incubated for further 24 h under permanent shaking at 130 rpm in a Multitron Standard incubation shaker (Infors HT) in the dark in a horizontal position to facilitate product extraction into the organic phase before sampling of 100 μL *n*-dodecane. The *n*-dodecane samples were subjected to gas chromatographic (GC) analysis employing the Agilent *6890N* gas chromatograph equipped with a (5%-phenyl)-methylpolysiloxane *HP-5* column (length, 30 m; inside diameter, 0.32 mm; film thickness, 0.25 μm; Agilent Technologies) and a flame ionization detector (FID). The injector and FID temperatures were set to 240 and 250°C, respectively. Volumes of 4 μL (patchoulol) or 2 μL (valencene) of a sample were injected splitless, with helium as carrier gas. The column temperature was maintained at 100°C for 5 min, increased at 10°C/min to 180°C, and then at 20°C/min to 300°C. The signals of heterologously produced terpenoids were assigned to products by comparison of retention times to commercial references of (−)-patchoulol from Carbosynth (product code: FP09677, retention time: 13.47 min) and (+)-valencene obtained from Sigma Aldrich (product number: 75056, retention time: 11.22 min). In order to determine product titers in cultures, the effective transfer from producing cells into the organic phase was assessed as described in Supplementary Method section “Analysis of *n*-Dodecane-Mediated Sesquiterpenoid Extraction From Phototrophically Grown *R. capsulatus.*” Essentially, cells were disrupted, extracted with *n*-dodecane and products were quantified using calibration curves of the reference compounds, taking into account the specific transfer efficiencies of the sesquiterpenoids into the organic phase in this process.

## Results

### Modular Concept for Engineering Sesquiterpenoid Synthesis in *R. capsulatus*

To establish stringently controlled recombinant expression in *R. capsulatus* under phototrophic growth conditions, we first constructed vector pRhon5Hi-2 (Supplementary Figure S1). The new expression vector carries the P*_*nif*_* promoter of the *R. capsulatus nifHDK* operon encompassing the structural genes of the molybdenum-dependent-nitrogenase enzyme complex (Haselkorn, 1986) as an *Nhe*I/*Xba*I fragment (NCBI Genbank Accession MG208548 deposited by Özgür and coworkers). This promoter is considered to be strong and is known to be strictly repressed by NH_4_^+^ and oxygen (Kranz and Haselkorn, 1985; Masepohl et al., 2002), thereby enabling the induction of target gene expression under phototrophic conditions (due to the absence of molecular oxygen) and ammonium depletion. Like all other pRho-vectors (Katzke et al., 2010, 2012), pRhon5Hi-2 is further characterized by a broad-host range replicon, a MOB site for conjugational transfer, two antibiotic resistance genes for plasmid maintenance, a hexahistidine-tag encoding region and a multiple cloning site for directed insertion of target genes as described previously.

Since the availability of molecular oxygen is one of the major environmental factors for controlling intrinsic ICM formation, carotenoid biosynthesis and nitrogen fixation in *R. capsulatus*, we first analyzed co-induction of P*_*nif*_*-dependent EYFP reporter gene expression (Supplementary Figure S2) and spheroidene/spheroidenone formation (Supplementary Figure S3) in dependence on O_2_. To this end, the EYFP gene was cloned in the expression vector pRhon5Hi-2 and subsequently transferred to *R. capsulatus* wildtype strain SB1003. The resulting clones were cultivated either under standard photoheterotrophic (i.e., anaerobic condition and constant illumination) or various microaerobic conditions (i.e., heterotrophic growth in the dark). To gradually restrict the oxygen concentration of the cultivation medium under non-phototrophic conditions, increasing filling volumes (20–70 ml) were applied in 100 mL shake flasks. Samples of all cultures were subjected to fluorescence and western blot analyses. The results of the EYFP expression studies clearly demonstrated that pRhon5Hi-2-mediated gene expression is fully induced under phototrophic conditions (-O_2_) whereas increasing oxygen concentrations in the medium resulted in a step-wise reduction of P*_*nif*_* activity. In contrast, high oxygen concentrations (Supplementary Figure S2, 20 mL) or addition of 15 mM ammonium (Supplementary Figure S2) led to a complete repression of P*_*nif*_*-controlled gene expression. As expected, carotenoid accumulation accompanied by ICM formation can analogously be induced in *R. capsulatus* by applying oxygen-limited and phototrophic cultivation conditions (Supplementary Figure S3). In summary, phototrophic cultivation leads to a concerted and strong co-induction of intrinsic terpene biosynthesis and P*_*nif*_*-controlled gene expression and was thus used for the following experiments.

**FIGURE 1:**
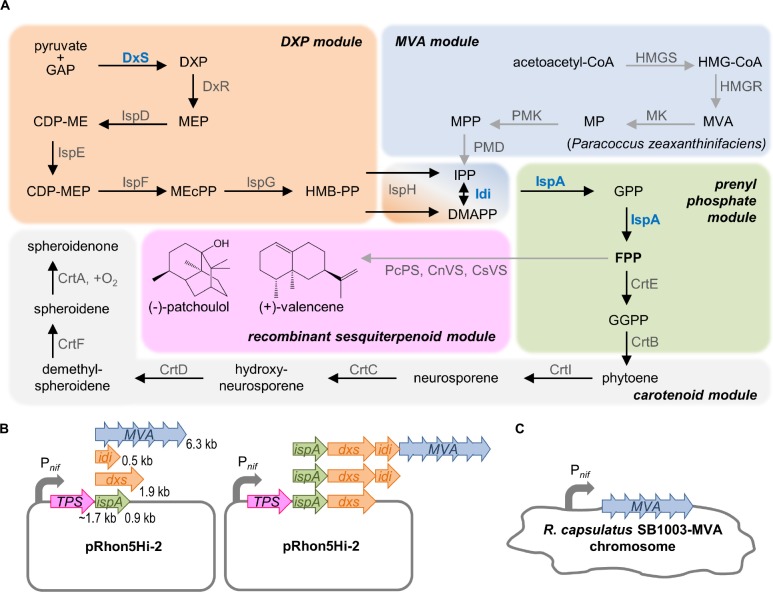
Strategy for modular engineering of sesquiterpenoid production in *R. capsulatus*. **(A)** The intrinsic terpene biosynthesis of *R. capsulatus* (black arrows) and implementation of recombinant terpene biosynthetic steps (gray arrows) is shown, schematically grouped in modules: The native DXP module, including the formation of name-giving DXP, synthesizes C5 building blocks IPP, and DMAPP from pyruvate and GAP. IPP and DMAPP are further naturally converted to elongated prenyl phosphates yielding GPP, FPP, and GGPP, and the latter represents the substrate for the formation of specific C40 carotenoid scaffolds of *R. capsulatus*. Recombinant sesquiterpenoid biosynthesis can be installed by conversion of FPP via respective terpene synthases (TPS) such as the patchoulol or valencene synthase. To increase the synthesis of plant sesquiterpenoids, the MVA pathway, including the formation of name-giving MVA, can be recruited as additional C5 building module. Here, *R. capsulatus* intrinsically uses acetyl-CoA to provide acetoacetyl-CoA, which can be converted to IPP/DMAPP by heterologous enzymes (of *Paracoccus zeaxanthinifaciens*). In addition, the DXP as well as the prenyl phosphate modules can be tuned by overexpression of typical bottleneck enzymes DxS, Idi and IspA (highlighted in blue). **(B)** Schematic representation of the plasmid system for the modular P*_*nif*_*-based co-expression of *ispA*, *dxs*, *idi* and/or the MVA gene cluster along with the TPS genes. Length of genes is indicated in kilo base pairs (kb). **(C)** Engineering of the *R. capsulatus* chromosome by integration of the MVA gene cluster in place of the *nifHDK* operon under control of P*_*nif*_*. See list of abbreviations.

In order to test the applicability of the P*_*nif*_* system for installing pathways of plant sesquiterpenoids (−)-patchoulol and (+)-valencene in *R. capsulatus*, the patchoulol synthase from *P. cablin* (PcPS) and the valencene synthase from *C. nootkatensis* (CnVS) were used. Both enzymes were previously described as highly active when expressed in the bacteria *Corynebacterium glutamicum* and *Rhodobacter sphaeroides* (Beekwilder et al., 2014; Binder et al., 2016; Henke et al., 2018). We also included the valencene synthase from *Citrus sinensis* (CsVS) which was previously reported to be less effective in this context (Beekwilder et al., 2014; Frohwitter et al., 2014) to comparatively evaluate the role of differential co-expression of precursor biosynthetic genes on variable sesquiterpenoid synthase-dependent FPP conversion. Based on engineering strategies that were previously identified as effective (Beekwilder et al., 2014; Khan et al., 2015), we aimed to establish an integrated concept of engineering modules that can be applied for recombinant sesquiterpenoid production in *R. capsulatus* (Figure 1).

FPP is the direct precursor for the production of heterologous sesquiterpenoids. Therefore, the strategies to increase the sesquiterpenoid production are primarily focused on an increased FPP supply, which can be achieved by enhancing the biosynthesis of FPP via overexpression of upstream pathway genes of the DXP, MVA, and prenyl phosphate modules.

To increase FPP biosynthesis in *R. capsulatus*, three enzymes of the intrinsic DXP pathway that are generally known to be rate-limiting were overexpressed: (i) the DxS-synthase and isopentenyl pyrophosphate isomerase (Idi) of *R. sphaeroides* (DXP module) and (ii) the IspA from *R. capsulatus* (prenyl phosphate module). In each case, expression of additional gene copies should provide an enhanced respective enzymatic activity in addition to the intrinsic capacities of the host. Moreover, we employed the alternative MVA pathway to establish a second route from central metabolism to isoprenoid biosynthesis. To this end (iii) the MVA gene cluster from *Paracoccus zeaxanthinifaciens* encompassing the genes *mvaA, idi, hsc, mvk, pmk*, and *mvd* (MVA module) was co-expressed. The genes encode all necessary enzymes for the conversion of acetoacetyl-CoA, which is provided by *R. capsulatus*, to IPP/DMAPP (Hümbelin et al., 2002).

For the construction of pRhon5Hi-2-based expression plasmids, the plant terpene synthase (TPS) genes, whose sequences were adapted to the *R. capsulatus* codon usage, were first inserted into the vector pRhon5Hi-2 thereby building the backbone for subsequent modular cloning steps. To evaluate individual effects of IspA, DxS, and Idi as well as the MVA enzymes, the respective genes were cloned downstream of the TPS genes in the vector (Figure 1B and Supplementary Figure S1). Moreover, to evaluate cumulative effects, the synthetic operons were stepwise extended by incremental combinations of the DXP and/or prenyl phosphate module genes together with the genes of the MVA module. Since the size of the synthetic operons grows with each module gene, which can lead to an increased instability of recombinant plasmids, we additionally integrated the 6.3-kb MVA gene cluster in place of the chromosomally located *nifHDK* operon of *R. capsulatus* SB1003 thereby placing it under control of P*_*nif*_* promoter (Figure 1C).

Details about plasmid cloning and strain generation including an overview about the plasmid cloning strategy, nucleotide sequences of codon-adapted terpenoid synthase genes and a primer table are summarized in Supplementary Figure S1 and Supplementary Tables S1, S2.

### Engineering of PcPS-Mediated Patchoulol Production in *R. capsulatus* via Modular Co-expression of Precursor Pathway Genes

**FIGURE 2:**
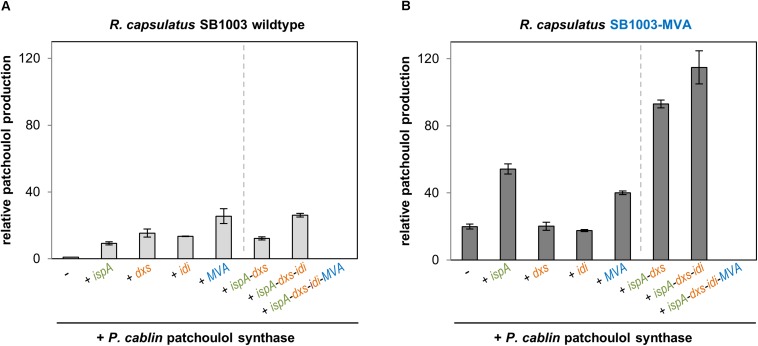
Patchoulol production in the *R. capsulatus* wildtype and modularly engineered strains. Patchoulol synthase PcPS from *Pogostemon cablin* was expressed in *R. capsulatus* SB1003 wildtype **(A)** and SB1003-MVA **(B)** harboring different sets of plasmid-encoded precursor module genes. Product formation was determined in cell cultures after 5 days of photoheterotrophic cultivation (late stationary growth phase). The sesquiterpene was sampled in an overlaid *n*-dodecane phase for GC-FID analysis. Increase of patchoulol production in engineered strains is shown as relative units using *R. capsulatus* SB1003 carrying the plasmid-encoded PcPS gene as reference strain. Data represent mean values and respective standard deviations from three independent cultivations. Plasmid-based co-expression of *ispA*, *dxs*, *idi*, and MVA genes is indicated below the bars. Strains with constructs for co-expression of the patchoulol synthase and single precursor module genes are grouped on the left side of the dashed line whereas strains with combined precursor module genes are shown on the right side.

To initially evaluate the applicability of *R. capsulatus* for the production of sesquiterpenoids, the expression plasmids pRhon5Hi-2-PcPS solely carrying the patchoulol synthase gene from *Pogostemon cablin* was first transferred into *R. capsulatus* wildtype strain SB1003. To analyze, if modular co-expression of different precursor genes (Figure 1) can help to increase patchoulol formation in the phototrophic host, plasmids pRhon5Hi-2-PcPS-ispA, pRhon5Hi-2-PcPS-dxs, pRhon5Hi-2-PcPS-idi and pRhon5Hi-2-PcPS-MVA (plasmids allowing co-expression of the patchoulol synthase together with a single precursor module) as well as pRhon5Hi-2-PcPS-ispA-dxs, pRhon5Hi-2-PcPS-ispA-dxs-idi and pRhon5Hi-2-PcPS-ispA-dxs-idi-MVA (i.e., plasmids with combined precursor module genes) were transferred into the same strain. To comparatively analyze patchoulol formation in all generated strains under phototrophic conditions, cells were cultivated anaerobically in an ammonium-depleted medium under constant illumination and product formation was determined in the late stationary growth phase by analyzing *n*-dodecane samples by use of gas chromatography (Figure 2).

Photoheterotrophic cultivation of *R. capsulatus* wildtype solely expressing *PcPS* led to a minor patchoulol signal (Figure 2A, see Supplementary Figure S4 for GC-MS analysis) which was absent in controls without the terpene synthase. In contrast, co-expression of IspA encoding *ispA* or DXP pathway genes *dxs* or *idi* resulted in an enhanced signal with a relative increase of factor 9–15. Co-expression of *PcPS* and the MVA gene cluster even increased the patchoulol accumulation 26-fold compared to the reference strain *R. capsulatus* SB1003 with pRhon5Hi-2-PcPS.

Next, we analyzed if patchoulol synthesis can be further enhanced in *R. capsulatus* when *PcPS* is co-expressed together with incremental combinations of respective precursor module genes. Surprisingly, none of the tested strains exhibited a significant increase of product formation. Remarkably, no patchoulol accumulation was detectable in the strain carrying the combination of all precursor module genes (*ispA-dxs-idi-MVA*) for plasmid-based expression. However, loss of the plasmid (18 kb) can be excluded as a reason for this finding due to positive selection conditions implemented with kanamycin in the medium. Further, instabilities of the promoter sequences or coding regions of isoprenoid converting enzymes appear unlikely since the same results could be observed in several independent experiments. Therefore, the results hint to feed-forward inhibition effects as further detailed in the discussion of this manuscript. In addition, we alternatively installed the MVA gene cluster of *P. zeaxanthinifaciens* in the *R. capsulatus* chromosome (Figure 1C).

To comparatively analyze sesquiterpenoid production in the recombinant strain *R. capsulatus* SB1003-MVA, the same pRhon5Hi-2-PcPS variants, that were previously evaluated in the wildtype strain SB1003, were used (Figure 2B). The expression of *PcPS* in *R. capsulatus* SB1003-MVA already led to a 20-fold increased patchoulol accumulation in comparison to the reference strain *R. capsulatus* SB1003 with plasmid pRhon5Hi-2-PcPS. Thus, in this strain patchoulol accumulates at comparable amounts that were gained in the respective wildtype strain harboring the plasmid-encoded MVA module. While co-expression of *PcPS* together with plasmid-encoded IspA, DxS or MVA module genes had moderate effects on product formation or did not lead to a further improved patchoulol production in *R. capsulatus* SB1003-MVA, the combined co-expression of *ispA-dxs* and *ispA-dxs-idi* resulted in remarkably higher product accumulation (93- and 115-fold in comparison to the reference strain). Again, no patchoulol accumulation was detectable with the plasmid carrying the assembly *ispA-dxs-idi-MVA*. In summary, we could show that in the *R. capsulatus* wildtype, the FPP precursor supply provided by the enzymes of the DXP, prenyl phosphate and MVA modules, represents a critical bottleneck for patchoulol production. Hence, a 126-fold increased patchoulol accumulation could be established in the phototrophic host upon concerted expression of chromosomally located MVA and plasmid-encoded *ispA, dxs*, and *idi* genes.

### CsVS- and CnVS-Mediated Valencene Production in *R. capsulatus* SB1003 and SB1003-MVA

By using the patchoulol synthase PcPS from *P. cablin*, we could demonstrate that the phototrophic bacterium *R. capsulatus* is basically suitable for the production of plant-derived sesquiterpenoids. In addition, our results clearly show that modular co-expression of homologous or heterologous enzymes involved in the supply of the central sesquiterpenoid precursor FPP can help to considerably increase product formation.

**FIGURE 3:**
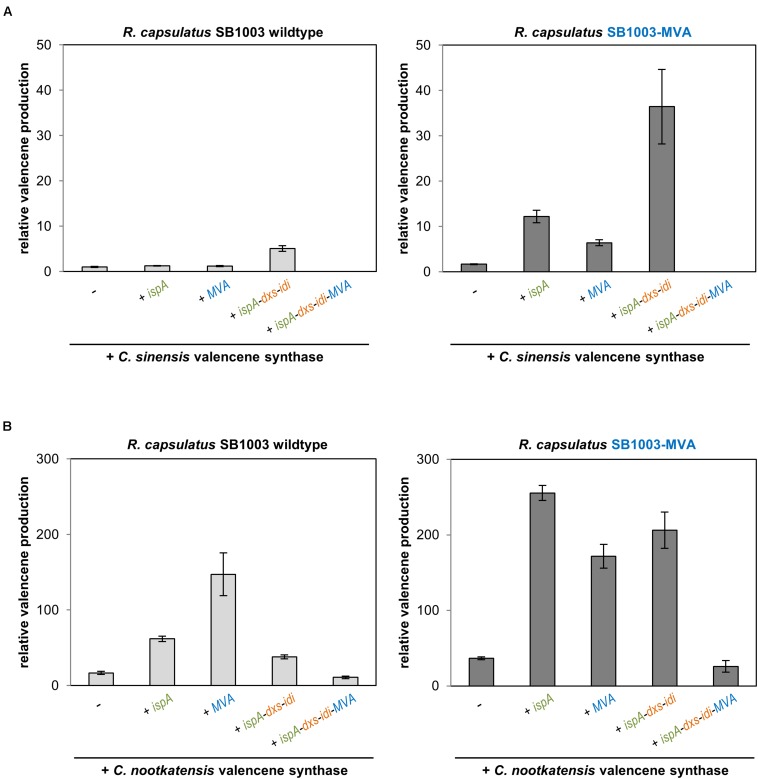
Valencene production in the *R. capsulatus* wildtype and engineered strains. Valencene synthases CsVS from *Citrus sinensis* **(A)** and CnVS from *Callitropsis nootkatensis* **(B)** were expressed using pRhon5Hi-2-derivatives harboring different sets of precursor module genes in *R. capsulatus* SB1003 wildtype and the engineered SB1003-MVA strain. Valencene was sampled after 5 days of photoheterotrophic cultivation in an overlaid *n*-dodecane phase for GC-FID analysis. Increase of valencene production in engineered strains is normalized to the peak area of the valencene signal of the respective reference strain (*R. capsulatus* wildtype strain SB1003 carrying the plasmid-encoded CsVS). Data represent mean values and respective standard deviations from three independent cultivations. Plasmid-based co-expression of *ispA*, *dxs*, *idi*, and MVA genes is indicated below the bars.

After these findings, we next investigated if the combination of precursor modules, which was identified as best for patchoulol production, can also be used for the synthesis of other plant sesquiterpenoids. To consider different activities of sesquiterpenoid synthases in this context, two valencene synthases from orange *Citrus sinensis* and Nootka cypress *Callitropsis nootkatensis* that were shown to perform differently in an *R. sphaeroides in vivo* assay (Beekwilder et al., 2014) were used for comparative studies in the modularly engineered *R. capsulatus* strains. To this end, the two codon-optimized valencene synthase genes were cloned into a set of pRhon5Hi-2 vectors carrying representative combinations of precursor module genes that had resulted in different patchoulol yields. The resulting plasmids were subsequently transferred into *R. capsulatus* wildtype strain SB1003 as well as the engineered strain SB1003-MVA. Cells were subsequently cultivated under photoheterotrophic conditions and valencene accumulation was comparatively analyzed in the late stationary phase (Figure 3).

As expected, expression of the CsVS gene in *R. capsulatus* SB1003 already resulted in accumulation of low but detectable amounts of valencene (Figure 3A and Supplementary Figure S4 for GC-MS analysis). In contrast to *PcPS*-mediated patchoulol production, co-expression of *CsVS* together with *ispA* or MVA genes only resulted in a marginal increase of product formation whereas concerted co-expression of *ispA*, *dxs* and *idi* led to a fivefold higher valencene amount. Expression of CsVS in *R. capsulatus* SB1003-MVA also resulted in a comparatively low valencene production (∼1.6 fold increase in comparison to the wildtype strain), while co-expression of IspA or the plasmid-encoded MVA module increased the sesquiterpenoid production by a factor of 12 and 6, respectively. However, combined co-expression of the *CsVS* gene together with *ispA*, *dxs* and *idi* in *R. capsulatus* SB1003-MVA provided remarkably higher product formation resulting in a 36-fold increase in relation to the reference strain SB1003 harboring pRhon5Hi-2-CsVS. The combination of all module genes (*ispA-dxs-idi-MVA*) for plasmid-based *CsVS* expression could not establish detectable valencene accumulation in the *R. capsulatus* wildtype and SB1003-MVA strain as previously found for patchoulol.

*C. nootkatensis* valencene synthase-dependent valencene synthesis was analogously tested in *R. capsulatus.* Here, expression of the alternative synthase gene in the *R. capsulatus* SB1003 wildtype already resulted in a 16-fold increased valencene accumulation (Figure 3B), thus corroborating previous studies reporting higher activity of CnVS compared to CsVS (Beekwilder et al., 2014; Frohwitter et al., 2014). Co-expression of *ispA* and plasmid-encoded MVA module further enhanced valencene accumulation levels up to 147-fold. Surprisingly, co-expression of *ispA* together with DXP module genes led to reduced valencene accumulation. The co-expression of all module genes (*ispA-dxs-idi-MVA*) further reduced product levels but notably yielded for the first time detectable product levels. Expression of *CnVS* in *R. capsulatus* SB1003-MVA already resulted in a 37-fold higher valencene accumulation compared to the reference strain. Remarkably, product formation could be further increased up to 255-fold by co-expression of *ispA*. In contrast to the results that were gained using the sesquiterpene synthases PcPS and CsVS, neither the implementation of the plasmid-encoded MVA pathway nor the co-expression of the DXP module genes resulted in a further increased product accumulation. These results clearly indicate that the implementation of new sesquiterpenoid synthases in the photosynthetic production host *R. capsulatus* requires, in any case, the evaluation of upstream pathway modules for gaining best results.

### Time-Dependent Patchoulol and Valencene Accumulation in Engineered *R. capsulatus* Strains

**FIGURE 4:**
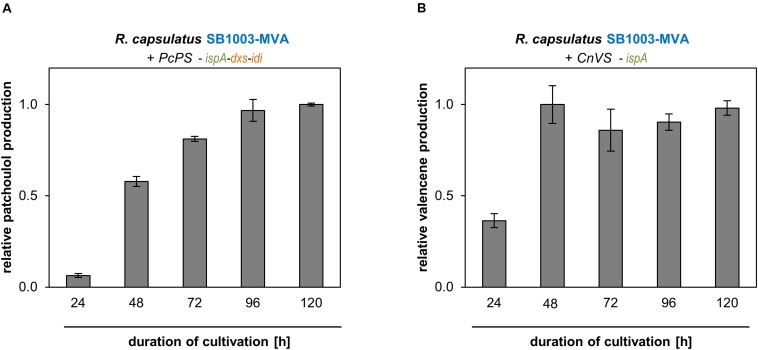
Patchoulol and valencene accumulation of best performing *R. capsulatus* strains over time. Patchoulol synthase PcPS from *P. cablin* **(A)** and valencene synthases CnVS from *C. nootkatensis* **(B)** were expressed in engineered *R. capsulatus* strains (as specified above panels). Relative product formation was determined during photoheterotrophic cultivation via GC-FID analysis of an overlaid *n*-dodecane phase. Data is normalized to highest peak area values, accordingly, and represents mean values and respective standard deviations from three independent cultivations.

Using patchoulol and valencene as showcase, we could demonstrate that *R. capsulatus* can be used as production host that accumulates plant-derived sesquiterpenoids during phototrophic growth. However, since we analyzed sesquiterpenoid formation so far only at the late-stationary growth phase, we subsequently performed an initial characterization of patchoulol and valencene production over time in strains that were determined to enable maximal product accumulation. Therefore, sesquiterpenoid production of *R. capsulatus* SB1003-MVA carrying vector pRhon5Hi-2-PcPS-ispA-dxs-idi and pRhon5Hi-2-CnVS-ispA, respectively, were monitored for 5 days (Figure 4).

In the logarithmic growth phase (24 h), patchoulol titers were comparatively low but steadily increased over incubation time until a maximum was reached after 96 h (Figure 4A). Low accumulation levels could initially also be observed for valencene production. However, in that case highest accumulation levels could already be detected after 48 h (Figure 4B). At this time-point, cells have typically reached the end of the exponential growth phase. After this, the valencene titer remained rather constant over time. To finally estimate product yields as accurately as possible, we analyzed (i) the individual transfer efficiencies of patchoulol and valencene from cell culture into the *n*-dodecane phase, (ii) the effect of the intracytoplasmic membrane that is predominantly formed by *R. capsulatus* under phototrophic conditions on *n*-dodecane-based product extraction, (iii) the differences in sesquiterpenoid transfer efficiencies after single compared to repeated *n*-dodecane extraction as well as, (iv) the effect of the *n*-dodecane layer on the final product titers (see Supplementary Method section “Analysis of *n*-Dodecane-Mediated Sesquiterpenoid Extraction From Phototrophically Grown *R. capsulatus*”). By taking the different factors into account, we were able to determine product titers of 24 ± 2 mg/L patchoulol (*R. capsulatus* SB1003-MVA with pRhon5Hi-2-PcPS-ispA-dxs-idi after 120 h of cultivation) and 18 ± 3 mg/L valencene (*R. capsulatus* SB1003-MVA harboring pRhon5Hi-2-CnVS-ispA after 48 h of cultivation), respectively. Corresponding productivities were calculated based on these values and the reached cell densities (Supplementary Table S3).

In summary, the results of *Rhodobacter*-based patchoulol and valencene production demonstrate that this bacterium can basically serve as an alternative sesquiterpenoid production chassis. In addition, the here presented P*_*nif*_*-based expression tools allow modular adaptation of precursor gene expression under phototrophic growth conditions and thereby tuning of sesquiterpenoid formation.

## Discussion

In this study we demonstrated the biosynthesis of plant sesquiterpenoids patchoulol and valencene in *R. capsulatus* under phototrophic conditions and the modular improvement of production by engineering of precursor biosynthesis. In particular, P*_*nif*_*-based co-expression of IspA, selected enzymes of the DXP pathway and the MVA pathway resulted in a substantial improvement of sesquiterpenoid production. The results are thus in agreement with previous studies, where engineering of isoprenoid precursor biosynthesis was shown to be a powerful means to boost terpenoid production in microbial hosts as recently reviewed by Schrader and colleagues for the production of terpenoid flavor and fragrance compounds (Schempp et al., 2018).

For example, the DXP synthase DxS in the intrinsic DXP pathway as well as the IPP isomerase Idi have previously been identified as central bottlenecks and their overexpression has been proven to be beneficial for bacterial isoprenoid production, e.g., in *E. coli* (Lv et al., 2013), *C. glutamicum* (Henke et al., 2018), and *R. capsulatus* (Khan et al., 2015). Increased DxS enzymatic capacity enhances the provision of the precursors IPP and DMAPP, which in turn can only be efficiently elongated if their ratio is adapted appropriately by IPP isomerase. While DMAPP and IPP are both required in the initial condensation of the two C5 units to C10 GPP, the further steps of elongation to C15 FPP and C20 GGPP specifically require additional IPP.

Although the DXP pathway is more prevalent in bacteria, a number of species additionally or exclusively possess the MVA pathway (Boucher and Doolittle, 2000). This route can be additionally implemented in bacterial producers, which are naturally equipped with only the DXP pathway, thereby further improving terpenoid precursor supply (e.g., Zurbriggen et al., 2012; Beekwilder et al., 2014). Here, we used MVA pathway genes from *P. zeaxanthinifaciens* for engineering *R. capsulatus*, as previous studies using the closely related bacterium *R. sphaeroides* indicated that these genes are suitable to boost terpenoid production in phototrophic α-proteobacteria (Beekwilder et al., 2014). *P. zeaxanthinifaciens* exclusively uses the MVA pathway (Eisenreich et al., 2002) which thus covers the entire cellular isoprenoid demand, e.g., for quinone and carotenoid formation (Boronat and Rodríguez-Concepción, 2015). The MVA genes from *P. zeaxanthinifaciens* are particularly suitable for the expression in *R. capsulatus* since both organisms exhibit an identical GC content (67%) which facilitates functional expression.

Besides the precursor pathways which provide the C5 units for isoprenoid synthesis, IspA activity is also of central importance for the production of sesquiterpenoids as this determines the available substrate pool for a given synthase enzyme. In line with this, multiple studies have previously described increased terpenoid production by co-expression of IspA in different hosts including *E. coli* for α-farnesene production (Wang et al., 2011) and *R. capsulatus* for squalene production (Khan et al., 2015).

Considering our findings of improved patchoulol and valencene titers upon P*_*nif*_* promoter-based co-expression of the mentioned genes, we corroborate here the usefulness of all three strategies, i.e., engineering of the DXP pathway, transfer of the MVA pathway, and reinforcing IspA activity, as well as synergies of their combination (Yang et al., 2016; Schempp et al., 2018). However, we could also observe negative effects of co-expressing precursor biosynthesis genes for sesquiterpenoid production. Such effects may be assigned to the fact that both, the DXP and the MVA pathway are strictly regulated to avoid accumulation of isoprenoid intermediates in the cell. Multiple regulatory circuits have been described to control the DXP pathway (Banerjee and Sharkey, 2014; Frank and Groll, 2017), including the feedback inhibition of DxS by IPP, and DMAPP (Banerjee et al., 2013), and feedback modulation of IspF activity by FPP (Bitok and Meyers, 2012). Similarly, the MVA pathway comprises known feedback loops. For example, HMG-CoA reductase and the MVA kinase are feedback inhibited by FPP (Miziorko, 2011; Scalcinati et al., 2012). Notably, accumulation of MVA pathway intermediates HMG-CoA and MVA, and likewise IPP and FPP were shown to exert toxic effects on *E. coli* and inhibit cellular growth (Martin et al., 2003; Pitera et al., 2007; Dahl et al., 2013). This effect can be alleviated by expression of heterologous terpene synthase enzymes. Prenyl phosphate elongation seems likewise controlled. IspA is inhibited by high concentrations of its substrate IPP and its product FPP, as it has been demonstrated for the human enzyme (Barnard and Popják, 1981; Park et al., 2017).

These phenomena indicate the central importance of the activity of an introduced sesquiterpenoid synthase. The enzyme’s capability to consume FPP most likely significantly determines which measures in the precursor biosynthetic pathways are beneficial, and which evoke adverse effects. In the present study, we therefore established a set of different expression vectors and an *R. capsulatus* expression strain SB1003-MVA equipped with the MVA pathway, so that different engineering strategies may be pursued and thereby, the metabolite flux can be adapted to different terpenoid synthases.

Scientific studies on the biotechnological production of patchoulol have largely focused on the yeast *S. cerevisiae* as host. There, engineering strategies have included expression of *P. cablin* patchoulol synthase together with IspA as fused protein and co-expression of HMG-CoA reductase of the MVA pathway, reaching 41.6 mg/L patchoulol (Gruchattka and Kayser, 2015). Yeast-derived patchoulol is already marketed by the company Firmenich. Recently, patchoulol production was installed in *C. glutamicum*, reaching a titer of 60 mg/L upon co-expression of *ispA*, *dxs* and *idi* in a fed-batch fermentation (Henke et al., 2018). We report here on 24 mg/L patchoulol production in photoheterotrophic cultivation of *R. capsulatus* carrying the MVA pathway genes in the chromosome and a plasmid for expression of *PcPS* along with *ispA*, *dxs* and *idi*. Valencene production has been established in diverse host systems, including *S. cerevisiae* (Beekwilder et al., 2014) and *Schizophyllum commune* (Scholtmeijer et al., 2014). A valencene titer of 41 mg/L could be achieved in *C. glutamicum* through strain optimization in combination with light-controlling gene expression (Binder et al., 2016). However, the highest titers of 352 mg/L were so far reached in an optimized *R. sphaeroides* strain (Beekwilder et al., 2014). Biotechnological valencene produced in yeast and *R. sphaeroides* is marketed by the companies Evolva and Isobionics, respectively. However, although modular engineering of the closely related *R. capsulatus* resulted in a 255-fold increased accumulation of valencene, the here reported titer is much lower (18 mg/L). Differences in product titers and accumulation over time clearly indicate that sesquiterpenoid production in *R. capsulatus* requires further investigation. Recently, it was demonstrated that yields of the sesquiterpene amorphadiene in chemoheterotrophically grown *R. sphaeroides* cells can be strongly increased by optimizing the cultivation conditions (Orsi et al., 2019). In this study, it could be shown that the C/N ratio, which can be altered by changing the supplemented carbon and nitrogen sources, as well as the oxygen availability, have an important impact on substrate-to-product conversion. Orsi et al. further speculated that poly-β-hydroxybutyrate (PHB), a storage compound which is formed in *Rhodobacter* under nitrogen-limiting conditions, can be utilized in the stationary growth phase thereby facilitating sesquiterpene production during growth limitation. Therefore, understanding the complex metabolic networks will be important to further improve the production of sesquiterpenes in phototrophic α-proteobacteria.

However, besides using *Rhodobacter* as a biotechnological production host, the specific physiological properties together with the here described modular adaptability of terpenoid formation make this bacterium an attractive candidate for future agricultural and therapeutic applications: (i) Secondary metabolites including sesquiterpenoids, which are produced by plants upon biotic stresses, are involved in direct and indirect plant defense mechanisms against herbivores and plant pathogens (Pichersky and Gershenzon, 2002; Wang et al., 2018; Block et al., 2019). While the role of terpenes in defense against aboveground plant pathogens and herbivores is well described, examples for belowground interspecies communication mechanisms are rare but indicate a crucial function of terpenes in plant defense against various pathogens including insects, fungi, bacteria and nematodes (Ohri and Pannu, 2009; Huang and Osbourn, 2019). In a preliminary study, we could now demonstrate that valencene is active against the plant pathogenic nematode *Heterodera schachtii* (Schleker et al. manuscript in preparation) – an observation that further corroborates this assumption. Since *R. capsulatus* was identified as a plant growth promoting bacterium naturally occurring in root microbiomes of various plants including *Brassica rapa*, rice, sugar beet, and barley (Çakmakçi et al., 2006, 2007; Gamal-Eldin and Elbanna, 2011; Hussein et al., 2014), appropriately engineered *Rhodobacter* strains delivering selected terpenes may constitute a possible future means for plant protection and reduction of fertilizer application in agricultural crop production. (ii) In addition, we could recently demonstrate that *R. capsulatus* can be used as an *in vivo* marker for multispectral optoacoustic tomography-based analysis of macrophage presence and activity inside of solid tumors (Peters et al., 2019). Therefore, production and reporter properties of *R. capsulatus* could also be combined to develop a new theranostic platform allowing selective *in situ* delivery of anti-cancer terpenoids together with visualization of the drug release process.

In summary, *R. capsulatus* poses a promising phototrophic host for the production of sesquiterpenoids. Besides these terpenoid targets, recombinant biosynthesis of carotenoids (tetraterpenoids) like β-carotene (Loeschcke et al., 2013) and the triterpenoids squalene, botryococcene, cycloartenol and lupeol in this bacterium has already been described (Khan et al., 2015; Loeschcke et al., 2017). However, the P*_*nif*_*-based expression tools together with the modular engineering approaches developed in this study can further help to characterize new terpenoids whose biosynthetic genes are identified via genome mining. In addition, it will facilitate the development of new sustainable production processes and chassis suitable for selective delivery of bioactive molecules. Indeed, ongoing studies in our group already indicate a broader applicability of this terpenoid production toolbox. Further refinement and dynamic control of precursor biosynthesis enabling plugging in different terpenoid pathways with individual precursor demands, and engineering of terpenoid secretion may contribute to improve sustainable terpenoid production.

## Data Availability

All data generated or analyzed during this study are included in the manuscript and/or the Supplementary Files.

## Author Contributions

TD and K-EJ conceived the research concept. KT, AL, and TD designed the experiments. KT, AÖ, VS, FH, SH, and TW performed the experimental work. BS-S performed the GC-MS analytics. KT, AL, JH-H, FG, SS, and TD analyzed the data and wrote the manuscript. All authors read and approved the final manuscript.

## Conflict of Interest Statement

The authors declare that the research was conducted in the absence of any commercial or financial relationships that could be construed as a potential conflict of interest.

## Abbreviations

CDP-ME: 4-diphosphocytidyl-2-*C*-methylerythritol
CDP-MEP: 4-diphosphocytidyl-2-*C*-methyl-D-erythritol 2-phosphate
CnVS: *Callitropsis nootkatensis* valencene synthase
CrtA: spheroidene monooxygenase
CrtB: phytoene synthase
CrtC: hydroxyneurosporene synthase
CrtD: hydroxyneurosporene desaturase
CrtE: GGPP synthase
CrtF: demethylspheroidene O-methyltransferase
CrtI: phytoene desaturase
CsVS: *Citrus sinensis* valencene synthase
DMAPP: dimethylallyl pyrophosphate
DXP: 1-deoxy-D-xylulose 5-phosphate
DxR: 1-deoxy-D-xylulose 5-phosphate reductoisomerase
DxS: 1-deoxy-D-xylulose 5-phosphate synthase
FPP: farnesyl pyrophosphate
GAP: glyceraldehydes-3-phosphate
GGPP: geranylgeranyl pyrophosphate
GPP: geranyl pyrophosphate
HMB-PP: (*E*)-4-Hydroxy-3-methyl-but-2-enyl pyrophosphate
HMG-CoA: 3-hydroxy-3-methylglutaryl-CoA
HMGR: 3-hydroxy-3-methyl-glutaryl-CoA reductase
HMGS: 3-hydroxy-3-methyl-glutaryl-CoA synthase
Idi: isopentenyl diphosphate isomerase
IPP: isopentenyl pyrophosphate
IspA: FPP synthase
IspD: 2-*C*-methyl-D-erythritol 4-phosphate cytidylyltransferase
IspE: 4-diphosphocytidyl-2-*C*-methyl-D-erythritol kinase
IspF: 2-*C*-methyl-D-erythritol 2,4-cyclodiphosphate synthase
IspG: (*E*)-4-hydroxy-3-methyl-but-2-enyl diphosphate synthase
IspH: (*E*)-4-hydroxy-3-methyl-but-2-enyl diphosphate reductase
MEcPP: 2-*C*-methyl-D-erythritol 2,4-cyclodiphosphate
MEP: 2-*C*-methyl-D-erythritol 4-phosphate
MK: mevalonate-5-kinase
MP: mevalonate-5-phosphate
MPP: mevalonate-5-pyrophosphate
MVA: mevalonate
PcPS: *Pogostemon cablin* patchoulol synthase
PMD: mevalonate-5-diphosphate decarboxylase
PMK: phosphomevalonate kinase.
