# Supplementary material for: Engineered Rhodobacter capsulatus as a Phototrophic Platform Organism for the Synthesis of Plant Sesquiterpenoids
Source: Front Microbiol. 2019 Sep 6;10:1998. doi: 10.3389/fmicb.2019.01998 (PMC6742980; doi:10.3389/fmicb.2019.01998)
Supplement: Supplementary file 1 [file Data_Sheet_1.pdf]

Supplementary Material

Engineered *Rhodobacter capsulatus* as a phototrophic platform organism for the synthesis of plant sesquiterpenoids

Katrin Troost[1], Anita Loeschcke[1,*], Fabienne Hilgers[1], Armagan Yakup Özgür[1], Tim Moritz Weber[1], Beatrix Santiago-Schübel[2], Vera Svensson[1], Jennifer Hage-Hülsmann[1], Samer S. Habash[3], Florian M. W. Grundler[3], A. Sylvia S. Schleker[3], Karl-Erich Jaeger[1,4], Thomas Drepper[1,*]

[1]Institute of Molecular Enzyme Technology, Heinrich Heine University Düsseldorf, Düsseldorf, Germany

[2]Central Institute for Engineering, Electronics and Analytics ZEA-3: Analytics, Forschungszentrum Jülich GmbH, Jülich, Germany

[3]INRES-Molecular Phytomedicine, Rheinische Friedrich-Wilhelms-University Bonn, Bonn, Germany

[4]Institute of Bio- and Geosciences IBG-1: Biotechnology, Forschungszentrum Jülich GmbH, Jülich, Germany

Content **page**

**1 Supplementary Figures.**		
**Supplementary Figure S1**	Construction scheme of pRhon5Hi-2-based expression vectors for sesquiterpenoid production in *R. capsulatus.*	2
**Supplementary Figure S2**	Oxygen-dependent control of P_{nif}-mediated gene expression in *R. capsulatus*.	4
**Supplementary Figure S3**	Oxygen-dependent control of intrinsic terpene formation in *R. capsulatus*.	5
**Supplementary Figure S4**	GC-MS analysis of (-)-patchoulol and (+)-valencene authentic references and products from recombinant *R. capsulatus*.	6
**2 Supplementary Methods.**		
**Supplementary Method**	Analysis of *n*-dodecane-mediated sesquiterpenoid extraction from phototrophically grown *R. capsulatus*.	8
**3 Supplementary Tables.**		
**Supplementary Table S1**	Codon optimized DNA sequences of plant genes for expression in *R. capsulatus*.	12
**Supplementary Table S2**	Oligonucleotides used in this study.	13
**Supplementary Table S3**	Production of patchoulol and valencene in *R. capsulatus* SB1003.	14

1 Supplementary Figures.

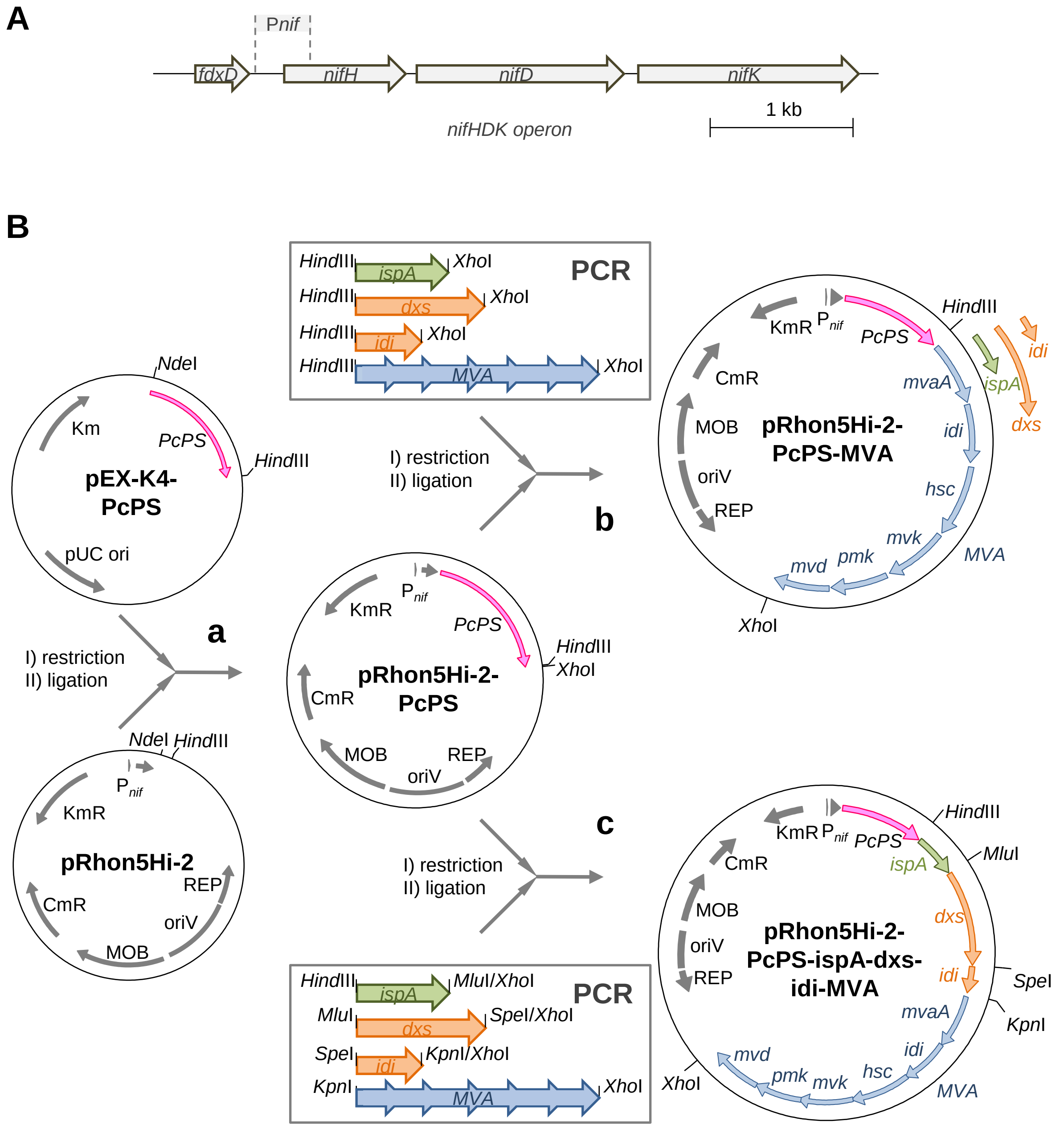

Supplementary Figure 1. Construction scheme of pRhon5Hi-2-based expression vectors for sesquiterpenoid production in *R. capsulatus*. (A) The *nifHDK* operon of *R. capsulatus* encodes the molybdenum dependent nitrogenase complex and comprises the structural genes *nifH* (dinitrogenase reductase), *nifD* and *nifK* (subunits of dinitrogenase). To exploit the phototrophic physiology of *R. capsulatus* for sesquiterpene production, we constructed the new expression plasmid pRhon5Hi-2 that carries the *nifHDK*-promoter region (P_{nif}): Dashed lines illustrate the genomic region which was

inserted as an *Nhe*I/*Xba*I fragment (NCBI Genbank Accession MG208548) into the respective sites of vector pRhotHi-2 (Katzke *et al*., 2010; doi:10.1016/j.pep.2009.08.008). As previously described, the broad-host range expression vector harbors two antibiotic resistance genes (chloramphenicol and kanamycin), an origin of replication (REP) and an origin of transfer (MOB). Target genes can be integrated into the multiple cloning site and are thereby placed under control of the P*nif* promoter. *fdxD*: Fe_2S_2 ferredoxin. **(B)** The scheme illustrates cloning steps for the example of patchoulol synthase PcPS from *Pogostemon cablin*. The synthase encoding gene was obtained as synthetic DNA fragment, flanked by recognition sequences for *Nde*I/*Hin*dIII, on a vector from Eurofins Genomics (pEX-K4-PcPS). The *PcPS* gene was isolated from the vector by use of *Nde*I/*Hin*dIII and ligated into likewise hydrolyzed expression vector pRhon5Hi-2 to yield pRhon5Hi-2-PcPS (**a**). Vectors pRhon5Hi-2-PcPS-ispA, pRhon5Hi-2-PcPS-dxs, pRhon5Hi-2-PcPS-idi and pRhon5Hi-2-PcPS-MVA were constructed by PCR-amplification of the respective genes with added recognition sequences for *Hin*dIII and *Xho*I in the primers, hydrolysis with these enzymes, and ligation of the fragments into likewise hydrolyzed pRhon5Hi-2-PcPS (**b**). The variant carrying the MVA gene cluster is depicted, while the other three alternative variants are conceptually indicated. For construction of vectors with incremental operons pRhon5Hi-2-PcPS-ispA, pRhon5Hi-2-PcPS-ispA-dxs, pRhon5Hi-2-PcPS-ispA-dxs-idi and pRhon5Hi-2-PcPS-ispA-dxs-idi-MVA, the genes were amplified with recognition sequences for different restriction endonucleases as depicted, added via the primers. Serial cloning of *ispA* as *Hin*dIII/*Xho*I fragment in the likewise hydrolyzed vector pRhon5Hi-2-PcPS, of *dxs* as *Mlu*I/*Xho*I fragment in the constructed pRhon5Hi-2-PcPS-ispA, of *idi* as *Spe*I/*Xho*I fragment in the constructed pRhon5Hi-2-PcPS-ispA-dxs and of the MVA gene cluster as *Kpn*I/*Xho*I fragment in the constructed vector pRhon5Hi-2-PcPS-ispA-dxs-idi yielded all constructs (**c**). Depicted is only the vector pRhon5Hi-2-PcPS-ispA-dxs-idi-MVA. KmR, *aphII* kanamycin resistence gene; CmR: chloramphenicol resistence gene; *ispA*, farnesyl pyrophosphate synthase gene from *R. capsulatus*; *dxs*, 1-deoxy-D-xylulose 5-phosphate synthase gene from *R. sphaeroides*; *idi*, isopentenyl pyrophosphate isomerase gene from *R. sphaeroides*; *MVA,* genes of the mevalonate biosynthesis pathway from *P. zeaxanthinifaciens.*

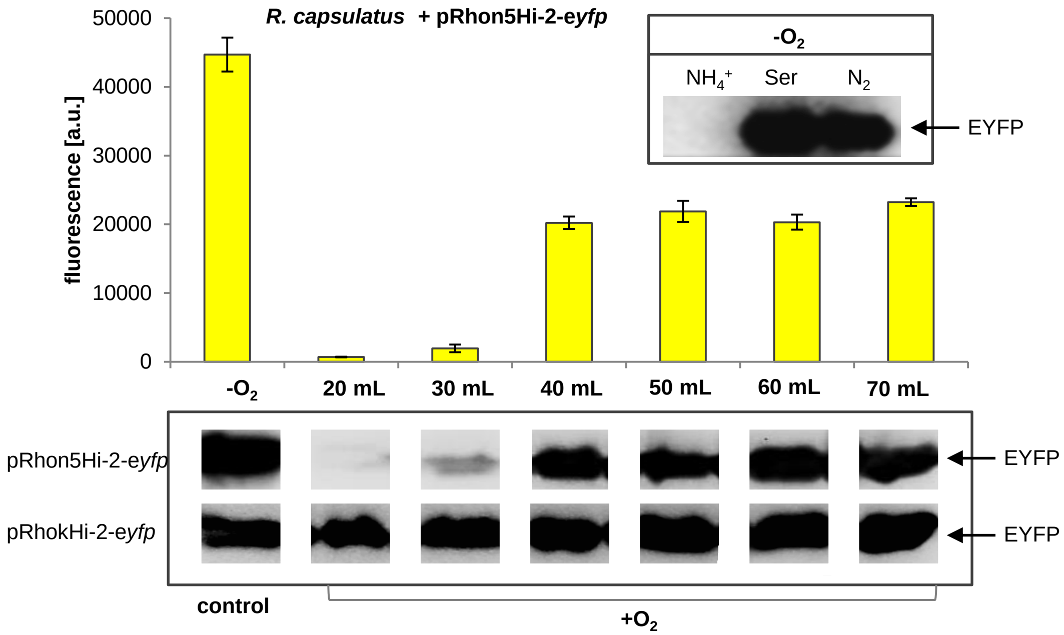

Supplementary Figure S2. Oxygen-dependent control of P_{nif}-mediated gene expression in *R. capsulatus*. Expression of the *eyfp* reporter gene was used as a measure to analyze the oxygen-sensitive P_{nif} promoter activity in *R. capsulatus* under different growth conditions. To this end, the reporter gene was cloned as an *Nde*I/*Hin*dIII fragment in the expression vector pRhon5Hi-2 under control of P_{nif}. Control cultures were cultivated under standard photoheterotrophic anaerobic conditions. **Upper panel:** *R. capsulatus* wildtype SB1003 carrying pRhon5Hi-2-*eyfp* was pre-cultivated in RCV medium with 0.1% ammonium, before test cultures were inoculated with an OD_{660nm} of 0.05 under aerobic conditions in 100 mL unbaffeled shake flasks (+O_2) with different filling volumes of RCV medium. Instead of ammonium, 0.1% serine was used as sole nitrogen source in order to exclusively control P_{nif} promotor activity via O_2 availability. After 48 h, cells equivalent to OD_{660nm} = 1 were harvested, re-suspended in SP-buffer (40 mM K_2HPO_4, 22 mM KH_2PO_4, 150 mM NaCl, 1 tablet of pH 8 Roche protease inhibitor per 250 mL; pH 7.2) and subjected to cell disruption using glass beads. Insoluble components of the extracts were pelleted and the supernatant containing soluble cellular proteins was incubated for 2 h before fluorescence analysis (excitation at 488 nm, emission 527 nm). Under phototrophic conditions (-O_2), cells exhibited the highest fluorescence signals, while samples from (micro)aerobic cultivation showed minor signals at 20 and 30 mL, and about 50% of the maximal values at 40 to 70 mL. Data represent mean values and respective standard deviations from three independent cultivations. **Lower panel:** To verify that fluorescence signals corresponded to protein expression, cell samples equivalent to OD_{660nm} = 1 were additionally subjected to SDS-PAGE and immunoblotting using antibodies *Anti-GFP* (Roche) and *Goat Anti-Mouse IgG (H+L)-HRP Conjugate* (BIO-RAD). A good correlation of protein bands with fluorescence signals was corroborated. To examine if results were indeed dependent on the promoter (and not otherwise physiology-related), protein expression was further evaluated in comparison to samples from likewise cultivated cells carrying an analogously cloned vector pRhokHi-2-*eyfp* (Katzke *et al*., 2010; doi:10.1016/j.pep.2009.08.008), where the reporter gene is under control of the constitutive P_{aphII} promoter. To further demonstrate the regulation of the P_{nif} promoter in dependence on the available nitrogen source, the EYFP-encoding reporter gene was expressed in *R. capsulatus* cells, where the respective test cultures were cultivated anaerobically

either with ammonium (NH_4^+; P_{nif} repressing conditions), or serine, or dinitrogen (Ser, N_2; P_{nif} derepressing conditions), as sole nitrogen sources in the medium (inset in upper panel).

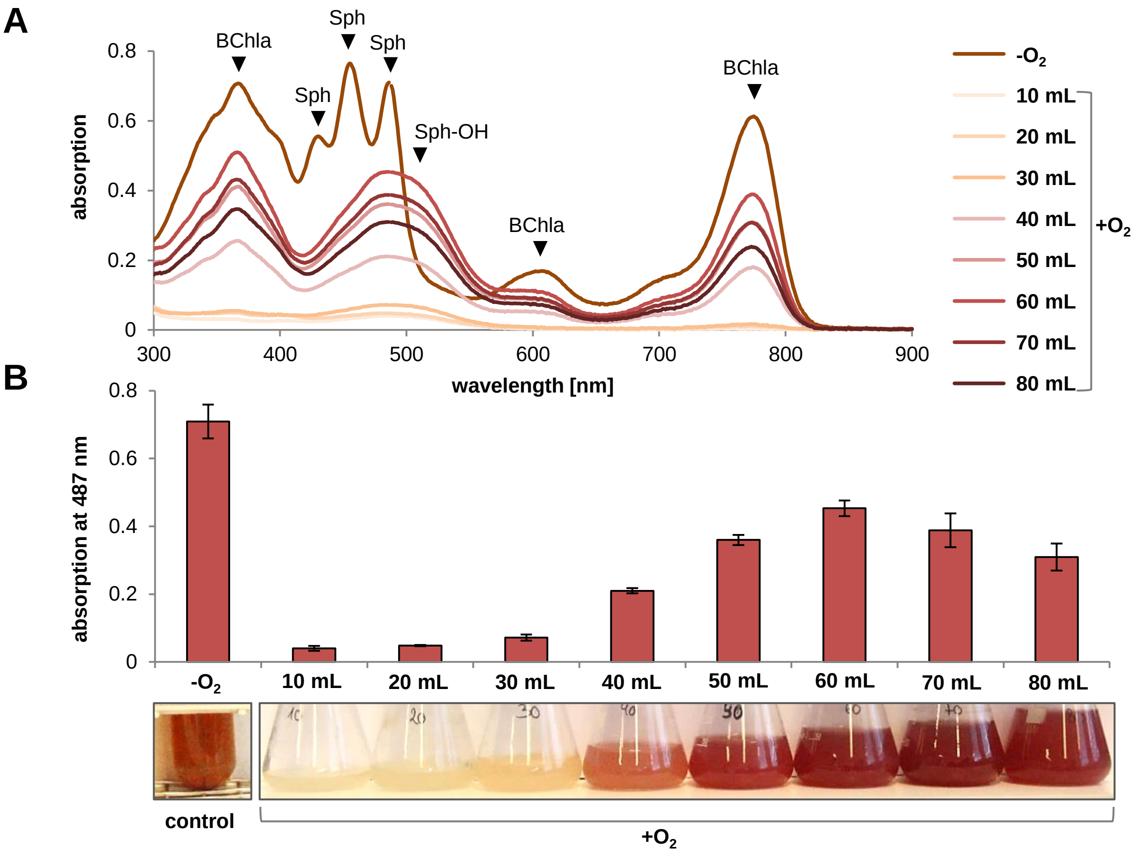

Supplementary Figure S3. Oxygen-dependent control of intrinsic terpene formation in *R. capsulatus*. Carotenoid formation was used as a measure to inspect the oxygen-sensitive intrinsic isoprenoid metabolism of *R. capsulatus* under different growth conditions. Control cultures were cultivated under standard photoheterotrophic anaerobic conditions. **(A)** *R. capsulatus* wildtype SB1003 was pre-cultivated in RCV medium with 0.1% ammonium, before test cultures were started with an OD_{660nm} of 0.05 under aerobic conditions in 100 mL unbaffeled shake flasks (+O_2) with different filling volumes of RCV medium supplemented with 0.1% serine. After 48 h, cells equivalent to OD_{660nm} = 1 were harvested and extracted with ethanol. Cell debris was pelleted and the supernatant containing cellular pigments was used for recording absorbance spectra from 300 to 900 nm in 1 nm intervals. Samples of phototrophically cultivated cells (-O_2) exhibited typical absorption maxima of bacteriochlorophyll *a* (BChla; 368, 600, and 770 nm), as well as characteristic maxima of the carotenoid spheroidene (Sph; 430, 456, and 487 nm). Under (micro)aerobic conditions, the same bacteriochlorophyll *a*-related absorption was detected (at lower levels), and the carotenoid-specific absorption of spheroidenone (Sph-OH; max. ~480 nm) was found, as expected. **(B)** Plotting the absorption at the wavelength of 478 nm in all samples showed that the pigment formation increased in +O_2 cultures with higher filling volumes up to 60 mL. Under this condition, about 65% of carotenoid-dependent absorption was reached compared to the photoheterotrophic anaerobic control. Data represent mean values and respective standard deviations from three independent cultivations.

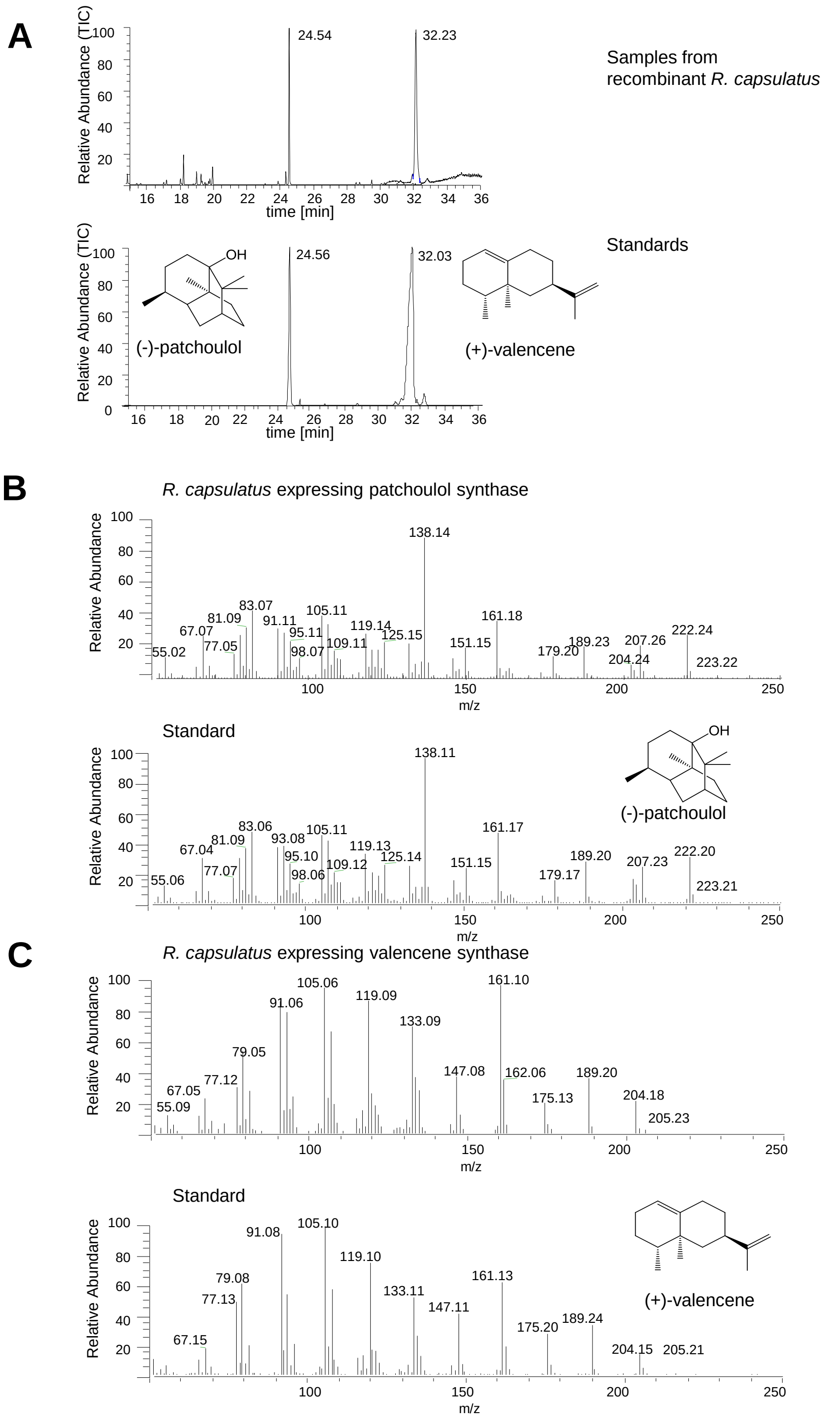

Supplementary Figure S4. GC-MS analysis of (-)-patchoulol and (+)-valencene from samples of recombinant *R. capsulatus*. (A) GC-MS chromatograms of (-)-patchoulol and (+)-valencene from recombinant *R. capsulatus* and the corresponding standards showing the same retention times. **(B)** EI-MS spectra of (-)-patchoulol from recombinant *R. capsulatus* and the corresponding standard at 24.5 min. **(C)** EI-MS spectra of (+)-valencene from recombinant *R. capsulatus* and the corresponding standard at 32.2 min. For GC-MS analysis, 1 µL of the samples was injected directly

into a Trace GC Ultra gas chromatograph coupled to ITQ 900 mass spectrometer (Thermo Scientific). Separation was achieved in a 30 m × 0.25 mm diameter capillary, with a 0.25 µm film of FS-5 supreme (CS Chromatographie Service). Split mode with a split ratio of 10 was used for the injector with the inlet temperature set to 250 °C. The oven was programmed to start at 100 °C and a 1 min hold, after which temperature increased to 300 °C at a rate of 5 °C/min. Helium was used as carrier gas and was adjusted to a flow rate of 1 mL/min. MS data were collected from 50 to 300 m/z during the temperature ramp.

2 Supplementary Methods.

Analysis of *n*-dodecane-mediated sesquiterpenoid extraction from phototrophically grown *R. capsulatus.*

Usually, sesquiterpenoids are extracted from microbial cell cultures via an *n*-dodecane layer (1/30 of the culture volume) which is added prior to cultivation and acts as an organic solvent phase (Rodriguez *et al.*, 2014; doi:10.1038/nprot.2014.132). In the here presented work, we used the photosynthetic bacterium *R. capsulatus* as alternative sesquiterpenoid production host. After phototrophic cultivation, the sealed Hungate tubes were shaken horizontally at 30 °C and 130 rpm overnight in the dark in a Multitron Standard incubation shaker (Infors HT) to facilitate sesquiterpenoid extraction into the organic phase. Subsequently, 100 µL *n*-dodecane samples were subjected to gas chromatographic (GC) analysis as described in the Materials and Methods section.
To quantify the final product titers, calibration curves with authentic references (-)-patchoulol and (+)-valencene were used. However, the mere correlation of signals from *n*-dodecane extracted samples with the reference signals does not take into account extraction efficiencies of individual sesquiterpenoids when using *n*-dodecane as organic solvent. It can be assumed that, in dependence of their specific properties, different sesquiterpenoids only diffuse to a certain extent into the *n*-dodecane layer. In addition, terpenes that are produced in the cytoplasm of *R. capsulatus* can additionally be retained by the intracytoplasmic membrane system thereby further affecting the transfer into the organic phase. Therefore, we first determined the transfer efficiency of valencene and patchoulol from cultivation medium into *n*-dodecane in the presence of intact and disrupted *Rhodobacter* cells. For this purpose, the respective reference compounds were first mixed with 14 mL of phototrophically grown *R. capsulatus* SB1003 cells (cultivation parameters: anaerobic growth, 30 °C, approx. up to OD_{660nm} = 2.5) in appropriate amounts (giving signal intensities comparable to samples from *R. capsulatus* production cultures; patchoulol: 15 mg/L; valencene: 5.71 mg/L). For this purpose, 130.4 µL valencene, which is an oil, was added as a 10-fold dilution in diethyl ether, while the solid patchoulol had to be solved in diethyl ether prior to use gaining a 2 mg/mL stock solution of which 110.5 µL was added. After addition, the cultures were sealed and vortexed for 1 min. Subsequently, reference substances were extracted using *n*-dodecane as described above. The transfer efficiency was determined via GC analysis by comparing peak areas of the specific signals from appropriately diluted solutions to samples that had undergone extraction (**Supplementary Figure S5**).

Supplementary Figure S5: Transfer efficiency of the patchoulol (black bar) and valencene (grey bar) reference compounds from cultivation medium into the *n*-dodecane phase in the presence of intact *R. capsulatus* cells. For extraction, 15 mg/L patchoulol or 5.71 mg/L valencene were added to 14 mL cell cultures (OD_{660nm} = 2.5). For details, see text above. Data represent means and standard deviations of three independent measurements (n = 3).

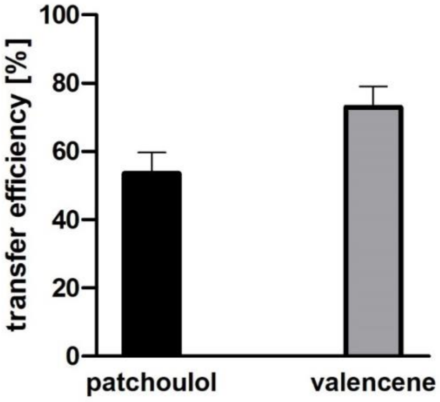

By using intact *R. capsulatus* cells, a transfer efficiency of 54% (patchoulol) and 73% (valencene) could be determined. Thus, it could be shown that there are some methodological losses, which have to be considered for product quantification.

To moreover analyze if putative interaction of intracellularly produced sesquiterpenoids with the *Rhodobacter* ICM can decrease product transfer, the experiment was repeated using disrupted cells. For this, equally cultivated *R. capsulatus* wildtype cells (OD_{660nm} = 2.5) were disrupted using a ball mill (3 x 10 min, 30 Hz, Mixer Mill MM 400, Retsch GmbH, Germany) and subsequently mixed with the same amount of reference compound as described previously. Extraction and quantification was performed as described for intact cell samples and signals were subsequently compared to those of the non-extracted reference compounds (**Supplementary Figure S6**).

Supplementary Figure S6: Transfer efficiency of the patchoulol (black bar) and valencene (grey bar) reference compounds from cultivation medium into the *n*-dodecane phase in the presence of disrupted *R. capsulatus* cells. For extraction, 15 mg/L patchoulol or 5.71 mg/L valencene were added to 14 mL cell lysate (OD_{660nm} = 2.5). For further details, see text above. Data represent means and standard deviations of three independent measurements (n = 3).

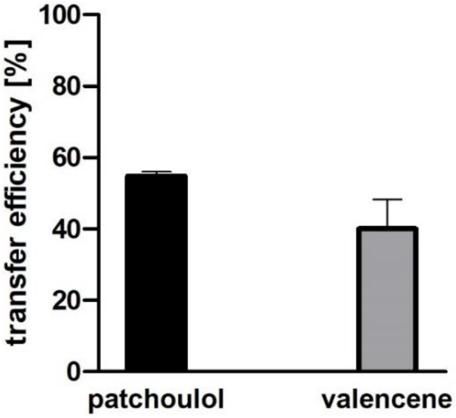

For patchoulol, no significant decrease of the transfer efficiency could be observed for lysed cells (55%) in comparison to the previous measurement using intact cells (54%). In contrast, a strong decrease of transfer efficiency could be detected for valencene (only 40%, in comparison to 73% when intact cells were used), suggesting that this more hydrophobic terpenoid (logP = 5.86 in

comparison to patchoulol with a logP of 4.19; values were calculated using the ALOGPS2.1 online tool described by Tetko *et al.* 2005; doi:10.1007/s10822-005-8694-y) can be retained more efficiently by the intracytoplasmic membrane system. Hence, for calculating the final production titers, individual transfer efficiencies for disrupted cell cultures (here termed c_t 'transfer efficiency coefficient'; patchoulol: 1.4521; valencene: 1.6) were taken into account.

Besides the above described negative effect of cellular components on the extraction efficiency, we further analyzed, if repeated *n*-dodecane-dependent sesquiterpenoid extraction should be considered for an optimal estimation of the overall production titers. Thus, an experiment with repeated sesquiterpenoid extractions from disrupted wildtype cultures that were mixed with reference compounds as described above was performed over four days (**Supplementary Figure S7**). For quantitative analysis of sesquiterpenoids, calibration curves with the authentic references of (-)-patchoulol and (+)-valencene ranging from 0.25 to 2 mg/500 µL *n*-dodecane, were used (slope: 380.37 and 475.37, respectively; see also depicted below in **Supplementary Figure S9**).

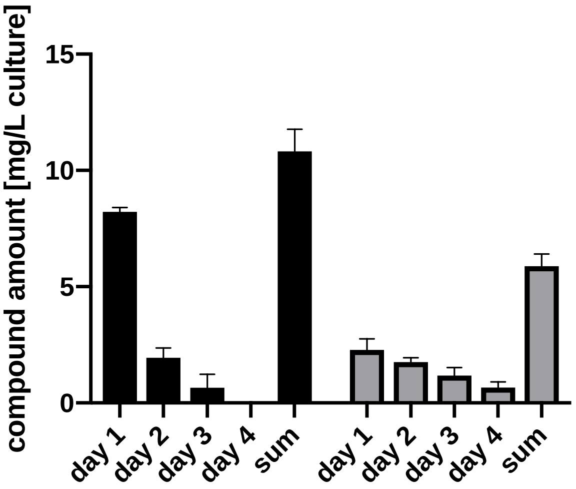

Supplementary Figure S7: Extraction efficiency of the patchoulol (black bars) and valencene (grey bars) reference compounds from cultivation medium in the presence of disrupted *R. capsulatus* cells by repeatedly using *n*-dodecane as organic solvent over four days. See text above for details. For repeated extraction, 15 mg/L patchoulol or 5.71 mg/L valencene were added to 14 mL cell lysate (OD_{660nm} = 2.5). Subsequently, 500 µL *n*-dodecane was used for 24 h over a time period of four days. Single extraction procedures were repeated four times and the sesquiterpenoid concentration of each fraction was analyzed via GC. Data represent means and standard deviations of three independent measurements (n = 3).

For patchoulol, the overall extraction efficiency was increased up to 32% by repeated extraction in comparison to the amount determined after the first extraction. For valencene, extraction efficiency increased even more (up to 157%). Therefore, we also took into account these factors by which the quantification on day 1 underestimates product titers (here termed c_{ex} 'coefficient for repeated extraction'; patchoulol: 1.3165; valencene: 2.5732 in order to calculate the final product titers.

Finally, we analyzed if the presence of an *n*-dodecane layer can positively or negatively affect sesquiterpene formation in *R. capsulatus* cells during cultivation. Therefore, an experiment with repeated sesquiterpene extraction out of production cultures that had been cultivated (5 days) with and without an *n*-dodecane layer before extraction was performed over four days (**Supplementary Figure S8**). For the analysis of sesquiterpene producing *R. capsulatus* cultures grown without a

solvent layer, equal amounts of *n*-dodecane were added after cultivation and prior to the extraction procedure.

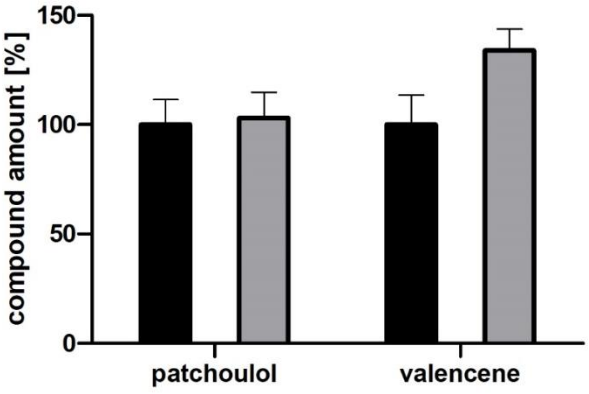

Supplementary Figure S8: Comparison of relative patchoulol and valencene formation in *R. capsulatus* production strains cultivated with (black bars) and without an *n*-dodecane layer (grey bars). Data was normalized to the amount of sesquiterpene extracted from cultures with *n*-dodecane. See text above for details. For *R. capsulatus* cultures containing the *n*-dodecane layer, 500 µL of the solvent was added before cultivation. In contrast, the same amount of solvent was added to cultures without *n*-dodecane after the cultivation. Compounds were extracted and analyzed as described above. Data represent means and standard deviations of three independent measurements (n = 3).

Almost no changes of sesquiterpene formation could be observed in the absence of the *n*-dodecane layer. Remarkably, production titers for valencene even increased slightly without using the organic solvent. Hence, the *n*-dodecane layer can alternatively be added after cultivation of the *Rhodobacter* production strains prior to the extraction procedure without any product losses.

In summary, product titers of *R. capsulatus* sesquiterpenoid production cultures were determined by analysis of *n*-dodecane extraction samples from disrupted cells. To this end, *R. capsulatus* strains were cultivated without the solvent, disrupted and then extracted one time with *n*-dodecane. Using the calibration curves obtained with reference compounds (**Supplementary Figure S9**) and taking into account above described results on losses of this procedure (**Supplementary Figure S6** and **S7**), we used the following equation for calculating the final patchoulol and valencene titers:

$$compound\ amount \left[\frac{mg}{L\ culture}\right] = \frac{pa}{s*v} * 1000\ mL * c_t * c_{ex}$$

$$pa = peak\ area\ [pA * s]$$

$$s = slope\ of\ calibration\ curve$$

$$v = culture\ volume\ [mL]$$

$$c_t = transfer\ efficiency\ coefficient$$

$$c_{ex} = coefficient\ for\ repeated\ extraction$$

Supplementary Figure S9: Quantification of extracted sesquiterpenes via calibration curves of patchoulol and valencene reference signals in GC-FID analyses. The signal intensities of the authentic reference compounds patchoulol **(A)** and valencene **(B)**, which were measured as peak areas [pA*s], were correlated to compound quantities [mg] in 500 µL-solutions with different concentrations. Mean values of detected signals of *n*-dodecane extraction samples from disrupted cells of the best *R. capsulatus* production strains (patchoulol: SB1003-MVA with pRhon5Hi-2-PcPS-ispA-dxs-idi, **(A)**; valencene: SB1003-MVA with pRhon5Hi-2-CnVS-ispA, **(B)**) are indicated (black lines from Y-axis to calibration line). Since extracts from *R. capsulatus* cultures were prepared with the same volume (500 µL) as were used for the dilutions of the reference compounds, the extracted amount of biosynthetic products [mg] could be deduced from the linear equations (indicated by dotted lines from calibration line to X-axis). These data were used for calculation of product levels in cultures, taking the used culture volume, and factors c_t and c_{ex} into account, as described above.

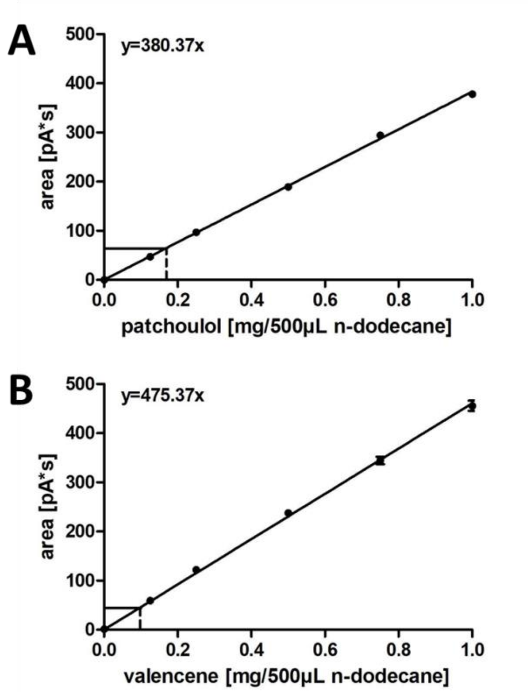

3 Supplementary Tables.

Supplementary Table S1. Codon optimized DNA sequences of plant genes for expression in *R. capsulatus*.

Patchoulol synthase PcPS from *Pogostemon cablin*

ATGGAACTGTATGCGCAGTCGGTGGGCGTGGGCGCCGCGTCGCGGCCGCTGGCGAACTTCCACCCCTGCGTGTGGGGCGATAAATTCATCGTCTATAACCCCCAGTCGTGCCAGGCCGGGGAG
CGGGAAGAGGCGGAAGAGCTGAAGGTCGAGCTCAAGCGGGAGCTGAAGGAAGCGAGCGATAACTATATGCGGCAGCTGAAAATGGTGGACGCCATCCAGCGCCTGGGGATCGATTATCTGT
TCGTCGAGGACGTCGATGAAGCCCTGAAGAACCTGTTCGAGATGTTCGATGCCTTCTGCAAAAACAACCATGACATGCATGCCACCGCCCTTTCCTTCCGGCTGCTGCGCCAGCATGGCTACCGC
GTCAGCTGCGAGGTCTTTGAGAAGTTCAAGGATGGGAAGGACGGCTTCAAGGTCCCGAACGAGGACGGCGCGGTCGCGGTGCTGGAATTTTTCGAAGCCACCCATCTGCGGGTCCACGGCGA
AGACGTGCTGGACAACGCCTTCGACTTCACGCGCAATTACCTTGAGTCCGTGTACGCCACCCTGAATGACCCGACCGCCAAACAGGTCCATAACGCGCTGAACGAGTTCTCGTTCCGCCGGGGC
CTGCCGCGCGTGGAAGCGCGCAAGTATATCTCCATCTATGAGCAATACGCGTCCCATCACAAGGGCCTTCTGAAGCTGGCGAAGCTGGACTTCAATCTGGTGCAAGCCCTGCATCGCCGCGAGC
TTTCCGAGGACTCGCGGTGGTGGAAAACCCTGCAAGTGCCCACGAAACTTTCGTTCGTGCGCGATCGGCTGGTCGAGTCGTACTTCTGGGCGTCCGGCTCGTACTTCGAGCCGAACTATAGCGT
CGCGCGCATGATCCTTGCGAAGGGGCTTGCCGTCCTGAGCCTGATGGATGATGTGTATGACGCGTATGGCACGTTTGAAGAGCTGCAGATGTTCACCGATGCGATCGAGCGCTGGGATGCGTC
CTGCCTGGACAAGCTGCCCGACTATATGAAAATCGTGTACAAAGCCCTTCTGGATGTCTTCGAAGAGGTCGACGAAGAACTGATCAAGCTGGGCGCGCCGTATCGCGCCTATTATGGGAAAGA
GGCGATGAAGTACGCGGCGCGGGCCTATATGGAGGAGGCCCAGTGGCGGGAGCAAAAACATAAGCCGACGACCAAAGAGTACATGAAGCTGGCCACCAAGACGTGCGGCTATATCACGCTG
ATCATCCTGTCGTGCCTGGGCGTGGAAGAAGGGATCGTCACGAAGGAAGCGTTCGATTGGGTGTTTTCCCGGCCGCCCTTTATCGAGGCGACCCTGATCATCGCCCGGCTGGTCAACGATATCA
CCGGCCATGAGTTCGAGAAGAAGCGCGAACATGTGCGCACCGCCGTGGAATGCTATATGGAGGAACATAAAGTCGGCAAGCAGGAGGTCGTGTCGGAGTTCTATAACCAGATGGAAAGCGCG
TGGAAGGATATCAATGAGGGCTTTCTGCGCCCCGTGGAATTCCCGATCCCGCTGCTGTACCTGATCCTTAACTCCGTGCGGACCCTGGAGGTGATCTATAAGGAAGGCGACTCGTATACCCATGT
GGGCCCCGCGATGCAAAACATCATCAAGCAGCTGTACCTTCACCCGGTGCCCTATTGA

Valencene synthase CsVS from *Citrus sinensis*

ATGTCCTCGGGCGAGACCTTCCGGCCCACCGCGGATTTCCATCCCTCCCTGTGGCGGAATCATTTCCTTAAAGGCGCCTCCGATTTCAAGACCGTGGATCACACCGCCACGCAAGAGCGGCACGA
AGCGCTGAAAGAGGAGGTCCGGCGGATGATCACGGATGCCGAAGACAAGCCGGTCCAAAAGCTGCGCCTGATCGACGAGGTCCAGCGCCTGGGCGTGGCGTACCACTTCGAGAAGGAAATC
GGCGACGCGATCCAAAAACTGTGCCCCATCTACATCGACTCCAACCGCGCCGACCTGCATACGGTGTCCCTGCACTTCCGCCTGCTGCGGCAGCAGGGCATCAAGATCTCGTGCGATGTCTTCGA
AAAGTTCAAAGACGACGAGGGGCGGTTCAAATCGTCGCTTATCAACGATGTGCAAGGGATGCTGAGCCTGTACGAAGCGGCGTATATGGCCGTCCGCGGCGAGCACATCCTGGATGAGGCGA
TCGCCTTTACGACCACCCATCTTAAATCGCTGGTCGCGCAAGACCATGTGACGCCCAAGCTGGCCGAACAGATCAACCACGCGCTGTACCGCCCGCTGCGGAAGACCCTTCCCCGCCTGGAGGC
GCGCTATTTTATGTCGATGATCAACAGCACGTCGGACCACCTGTGCAACAAGACCCTGCTGAATTTCGCGAAACTGGATTTTAATATCCTTCTGGAGCTGCATAAGGAAGAGCTGAACGAGCTGA
CCAAATGGTGGAAGGACCTGGATTTCACCACGAAGCTGCCGTATGCCCGGGATCGCCTGGTGGAACTTTACTTCTGGGATCTGGGCACCTACTTCGAACCGCAGTACGCCTTCGGGCGCAAAAT
CATGACGCAGCTGAACTACATCCTGTCGATCATCGACGACACGTACGATGCGTATGGCACCCTGGAAGAACTGAGCCTGTTTACCGAGGCGGTGCAGCGCTGGAATATCGAGGCGGTGGACAT
GCTGCCGGAGTACATGAAGCTGATCTACCGGACCCTGCTGGATGCCTTCAATGAAATCGAGGAGGATATGGCCAAGCAGGGGCGCTCGCACTGCGTGCGCTACGCCAAGGAAGAAAACCAGA
AAGTGATCGGCGCGTATTCGGTCCAAGCCAAGTGGTTCAGCGAGGGCTATGTCCCGACCATCGAAGAATACATGCCGATCGCGCTGACCAGCTGCGCGTATACCTTTGTCATCACCAATTCCTTC
CTGGGCATGGGCGATTTCGCGACCAAGGAGGTGTTCGAGTGGATCTCGAACAACCCGAAGGTGGTGAAGGCCGCCTCGGTCATCTGCCGGCTTATGGACGATATGCAGGGCCACGAGTTTGA
GCAGAAGCGCGGCCACGTGGCGAGCGCGATCGAATGCTATACCAAGCAACATGGCGTGTCGAAGGAGGAAGCCATCAAGATGTTCGAGGAGGAAGTGGCCAATGCCTGGAAGGACATCAAC
GAAGAACTGATGATGAAGCCGACCGTGGTGGCGCGCCCGCTGCTGGGCACCATCCTGAACCTGGCGCGGGCGATCGACTTCATCTACAAGGAAGATGATGGCTACACCCATAGCTATCTGATC
AAAGACCAAATCGCGTCCGTGCTGGGCGATCATGTGCCGTTCTGA

Valencene synthase CnVS from *Callitropsis nootkatensis*

ATGGCGGAAATGTTCAACGGCAACAGCAGCAACGATGGCAGCAGCTGCATGCCGGTGAAAGATGCGCTGCGCCGCACCGGCAACCATCATCCGAACCTGTGGACCGATGATTTCATCCAGAGC
CTGAACAGCCCGTATAGCGATAGCAGCTATCATAAACATCGCGAAATCCTGATCGATGAAATCCGCGATATGTTCAGCAACGGCGAAGGCGATGAATTCGGCGTGCTGGAAAACATCTGGTTCG
TGGATGTGGTGCAGCGCCTGGGCATCGATCGCCATTTCCAGGAAGAAATCAAAACCGCGCTGGATTATATCTATAAATTCTGGAACCATGATAGCATCTTCGGCGATCTGAACATGGTGGCGCT
GGGCTTCCGCATCCTGCGCCTGAACCGCTATGTGGCGAGCAGCGATGTGTTCAAAAAATTCAAAGGCGAAGAAGGCCAGTTCAGCGGCTTCGAAAGCAGCGATCAGGATGCGAAACTGGAAAT
GATGCTGAACCTGTATAAAGCGAGCGAACTGGATTTCCCGGATGAAGATATCCTGAAAGAAGCGCGCGCGTTCGCGAGCATGTATCTGAAACATGTGATCAAAGAATATGGCGATATCCAGGA
AAGCAAAAACCCGCTGCTGATGGAAATCGAATATACCTTCAAATATCCGTGGCGCTGCCGCCTGCCGCGCCTGGAAGCGTGGAACTTCATCCATATCATGCGCCAGCAGGATTGCAACATCAGC
CTGGCGAACAACCTGTATAAAATCCCGAAAATCTATATGAAAAAAATCCTGGAACTGGCGATCCTGGATTTCAACATCCTGCAAAGCCAGCATCAGCATGAAATGAAACTGATCAGCACCTGGT
GGAAAAACAGCAGCGCGATCCAGCTGGATTTCTTCCGCCATCGCCATATCGAAAGCTATTTCTGGTGGGCGAGCCCGCTGTTCGAACCGGAATTCAGCACCTGCCGCATCAACTGCACCAAACT
GAGCACCAAAATGTTCCTGCTGGATGATATCTATGATACCTATGGCACCGTGGAAGAACTGAAACCGTTCACCACCACCCTGACCCGCTGGGATGTGAGCACCGTGGATAACCATCCGGATTATA
TGAAAATCGCGTTCAACTTCAGCTATGAAATCTATAAAGAAATCGCGAGCGAAGCGGAACGCAAACATGGCCCGTTCGTGTATAAATATCTGCAAAGCTGCTGGAAAAGCTATATCGAAGCGTA
TATGCAGGAAGCGGAATGGATCGCGAGCAACCATATTCCGGGCTTCGATGAATATCTGATGAACGGCGTGAAAAGCAGCGGCATGCGCATCCTGATGATCCATGCGCTGATCCTGATGGATAC
CCCGCTGAGCGATGAAATCCTGGAACAGCTGGATATCCCGAGCAGCAAAAGCCAGGCGCTGCTGAGCCTGATCACCCGCCTGGTGGATGATGTGAAAGATTTCGAAGATGAACAGGCGCATGG
CGAAATGGCGAGCAGCATCGAATGCTATATGAAAGATAACCATGGCAGCACCCGCGAAGATGCGCTGAACTATCTGAAAATCCGCATCGAAAGCTGCGTGCAGGAACTGAACAAAGAACTGCT
GGAACCGAGCAACATGCATGGCAGCTTCCGCAACCTGTATCTGAACGTGGGCATGCGCGTGATCTTCTTCATGCTGAACGATGGCGATCTGTTCACCCATAGCAACCGCAAAGAAATCCAGGAT
GCGATCACCAAATTCTTCGTGGAACCGATCATCCCGTAA

Supplementary Table S2. Oligonucleotides used in this study.

Name	Application	Nucleotide sequence (5‘→3‘)
**MVA gene cluster interposon vector**		
Nif up for NdeI	PCR of *nifHDK* upstream region	ATATACATATGGGATCAGCCGGTTGATCAGC
Nif up rev KpnI-XbaI	PCR of *nifHDK* upstream region	ATATAGGTACCATCGATCCATCTAGACGGCCAGGTGCAGC ACGGTG
Nif dwn for XbaI-KpnI	PCR of *nifHDK* downstream region	TATATTCTAGATGGATCGATGGTACCCAAGGCGACGCTCT CGATGC
Nif dwn rev EcoRI-XhoI	PCR of *nifHDK* downstream region	TATATGAATTCCTCGAGGTCTGCGCGAGTTCCATGGG
MVA for XbaI	PCR of MVA gene cluster	ATATATCTAGAAATAATTTTGTTTAACTTTAAGAAGGAGATA TACATATGATGCAGAACGAAGAAGC
MVA rev KpnI NheI	PCR of MVA gene cluster	ATATAGGTACCATATAGCTAGCTCAACGCCCCTCGAACGG CG
GmR for NheI SpeI	PCR of aacC1 gene	ATATAGCTAGCACTAGTGACGCACACCGTGGAAACG
GmR rev KpnI	PCR of aacC1 gene	ATATAGGTACCGTTAGGTGGCGGTACTTGGG
mobTc for XhoI	PCR of oriT-Tet	TATATCTCGAGAAGCGAGCCAGCCGGTGG
mobTc rev XhoI	PCR of oriT-Tet	TATATCTCGAGCGAGGTGCCGCCGGCTTC
**Construction of expression vector pRhonHi-2**		
Pnif-fw	PCR of *nifHDK* promoter region	AATCGCTAGCTCCCGACAGAGGG
Pnif-rv	PCR of *nifHDK* promoter region	CGATTCTAGACGGCCAGGTGCA
**Individual co-expression of precursor biosynthetic genes**		
HindIII*ST_ispA fw	PCR of *ispA* gene	ATTAAGCTTTGAAAGAAGGAGATATAATGTTTTCCGAACGT TTG
XhoI_ispA rv	PCR of *ispA* gene	AATACTCGAGCTTGTCGCGTTCGATCACATAG
HindIII*ST_dxs fw	PCR of *dxs* gene	ATTAAGCTTTGAAAGAAGGAGATATAATGACCGACAGACC CTGC
XhoI_dxs rv	PCR of *dxs* gene	AATACTCGAGTCAGGCGCGGCGGGCGAG
HindIII*ST_idi fw	PCR of *idi* gene	ATTAAGCTTTGAAAGAAGGAGATATAATGACGGAAATGGT TCCCGC
XhoI_idi rv	PCR of *idi* gene	AATACTCGAGTTACTGCACGACGCGCAGC
HindIII*ST_MVA fw	PCR of MVA gene cluster	ATTAAGCTTTGAAAGAAGGAGATATAATGATGCAGAACGA AGAAGC
MVA rev XhoI	PCR of MVA gene cluster	ATATACTCGAGTCAACGCCCCTCGAACG
**Co-expression of incremental operons of precursor biosynthetic genes**		
HindIII*ST_ispA fw	PCR of *ispA* gene	ATTAAGCTTTGAAAGAAGGAGATATAATGTTTTCCGAACGT TTG
ispA rev XhoI MluI	PCR of *ispA* gene	ATATACTCGAGATATAACGCGTTCACTTGTCGCGTTCGAT CAC
dxs for MluI	PCR of *dxs* gene	ATATAACGCGTATAAGGAGATATACACATGACCGACAGAC CCTGC
dxs rev XhoI SpeI	PCR of *dxs* gene	ATATACTCGAGATATAACTAGTTCAGGCGCGGCGGGCGA G
idi for SpeI	PCR of *idi* gene	ATATAACTAGTGCCTTTGACAAGGAATTGAATGACGGAAAT GGTTCCCGC
idi rev XhoI KpnI	PCR of *idi* gene	ATATACTCGAGATATACCATGGTTACTGCACGACGCGCAG C
MVA for KpnI	PCR of MVA gene cluster	ATATACCATGGTCTAGAAATAATTTTGTTTAAC
MVA rev XhoI	PCR of MVA gene cluster	ATATACTCGAGTCAACGCCCCTCGAACG

Supplementary Table S3. Production of patchoulol and valencene in *R. capsulatus* SB1003.

Compound	Patchoulol	Valencene
***Rhodobacter capsulatus* strain**	**SB1003-MVA +PcPS-ispA-dxs-idi**	**SB1003-MVA +CnVS-ispA**
Titer at below defined time point (mg/L culture)	24±2 mg/L	18±3 mg/L
Time point of highest titer (h)	120 h	48 h
OD at the time point of highest titer (660 nm)	3.02±0.10	2.79±0.06
Cell mass at the time point of highest titer (gDCW/L culture)	1.81±0.06 gDCW/L	1.67±0.03 gDCW/L
Volumetric productivity (mg/L/h)[1]	0.20±0.02 mg/L/h	0.38±0.06 mg/L/h
Specific yield at above defined time point (mg/gDCW)	13.2±0.8 mg/gDCW	10.8±1.6 mg/gDCW
Specific productivity (mg/gDCW/h)[1]	0.11±0.01 mg/gDCW/h	0.22±0.03 mg/gDCW/h

[1]Productivities per hour were calculated based on product levels that were present at the time points when highest titers were reached, and are thus not necessarily maximal productivities.
